# Quantitative video analysis of head acceleration events: a review

**DOI:** 10.3389/fbioe.2025.1658222

**Published:** 2025-08-20

**Authors:** Thomas Aston, Filipe Teixeira-Dias

**Affiliations:** Institute for Infrastructure and Environment (IIE), School of Engineering, The University of Edinburgh, Edinburgh, United Kingdom

**Keywords:** head acceleration events, contact sports, videogrammetry, computer vision, sports medicine, concussion, neurodegenerative disease

## Abstract

The biomechanics of head acceleration events (HAEs) in sport have received increasing attention due to growing concern over concussion and long-term neurodegenerative disease risk. While wearable sensors, such as instrumented mouthguards (iMGs), are now commonly used to measure HAEs, these devices face well-documented challenges, including poor skull coupling, limited compliance, and high false-positive rates. Video footage is routinely collected in sports for performance analysis, and is a perhaps underutilised source for both retrospective and *in situ* measurement surrounding HAEs. Traditionally used to confirm HAE exposure in wearable sensor studies, video has more recently been explored as a quantitative tool in its own right. This review synthesises the current state of the art in video-based measurement of HAEs, with a particular focus on videogrammetric methods, including manual point tracking and model-based image matching. Recent advances in computer vision and deep learning that offer the potential to automate and extend these approaches are also examined. Key limitations of current video-based methods are discussed, alongside opportunities to improve their scalability, accuracy, and biomechanical insight. By consolidating evidence across traditional and emerging approaches, this review highlights the potential of video as a practical and valuable measurement source for quantitative measurement and modelling of HAEs in sport.

## 1 Introduction

The high-speed and collision-intensive nature of contact sports such as association football (or soccer, globally), rugby, and American football exposes athletes to frequent head acceleration events (HAEs) (Tierney, 2021; Tooby et al., 2024), contributing to high rates of sports-related concussion (SRC) (Daneshvar et al., 2011; Daniel et al., 2012) and an increased risk of long-term neurodegenerative diseases (NDs), including chronic traumatic encephalopathy (CTE) (Mackay et al., 2019; McKee et al., 2009; Russell et al., 2021; 2022; Stewart et al., 2023). These concerns have attracted widespread media coverage and public attention, including through high-profile legal actions, such as the $765 million settlement between the NFL and former players in 2013 (Belson, 2013), the class-action lawsuit filed against the NHL (Kaplan, 2021), and ongoing legal proceedings in the UK involving the Rugby Football Union (RFU) and Rugby Players Association (RPA) (Bull, 2024). Going forward, there is therefore a clear need for improved understanding and monitoring of HAEs to inform preventative strategies and provide better protection to athletes across all levels of play.

The precise mechanisms by which SRC and HAE exposure contribute to ND risk remain unknown. The most recent report of the Lancet Commission included head injury as a modifiable risk factor for dementia for the first time (Livingston et al., 2020). In an attempt to quantify the burden of HAEs, recent efforts in the biomedical and biomechanical engineering fields have seen the adoption of wearable sensors for field-based HAE monitoring (Le Flao et al., 2022; O’Connor et al., 2017), and advanced computational modelling strategies to quantify brain injury risk (Ji et al., 2022). However, wearable devices have several limitations, including sensor-skull coupling issues (Wu et al., 2016b), varying accuracy with proximity of impact to sensor (Le Flao et al., 2025), user compliance challenges (Jones et al., 2022; Kenny et al., 2024; Roe et al., 2024), and the need for extensive manual video confirmation to verify true positives (Kuo et al., 2018; Patton et al., 2020).

Video, however, is already widely used in sport for performance analysis and incident review, and offers a low-cost, non-invasive means of collecting exposure data retrospectively (Funk et al., 2022; Tierney et al., 2019). When combined with appropriate analysis techniques, video footage can support both qualitative assessments (e.g., verifying impact events or classifying impact scenarios (Rotundo et al., 2023; Sherwood et al., 2025)) and quantitative measurements of head motion and impact mechanics (Bailey et al., 2018; Gyemi et al., 2023; Stark N. E.-P. et al., 2025), making it increasingly valuable in HAE research.

Nevertheless, the use of video for quantitative measurement remains underdeveloped. Traditional videogrammetric methods, including point tracking and model-based image matching (MBIM), have been validated for measuring impact velocities in controlled environments (Bailey et al., 2018; Tierney et al., 2018a). However, they often rely on high-speed cameras, multiple calibrated views, and considerable manual effort, limiting their scalability in field settings (Stark N. E.-P. et al., 2025; Tierney et al., 2019). These constraints have historically prevented widespread deployment in large-scale epidemiological studies. More recently, however, advances in computer vision and deep learning, such as human and head pose estimation (Asperti and Filippini, 2023; Marchand et al., 2016) and action detection (Giancola et al., 2023; Rezaei and Wu, 2022), have shown potential as approaches for automating the process of extracting motion data from standard broadcast or handheld video. These developments present new opportunities for applying video-based HAE analysis in both research and clinical settings.

This narrative review synthesises the literature on quantitative videogrammetric methods applied to consumer-grade video footage for analysing head, body and object motion resulting from HAEs. The focus is on approaches that estimate measurable kinematic parameters associated with HAEs, including positions, velocities, orientations, and trajectories. Emphasis is placed on both traditional methods and emerging deep learning solutions, evaluating their current accuracy, scalability, and relevance to broader HAE research interests.

The remainder of this review is structured as follows. Section 2 introduces the research areas associated with measurement of HAEs, highlighting the expanding role of quantitative video analysis across the full spectrum of HAE research, and the associated challenges that come with it. This includes not only direct estimation of head kinematics, but also broader contextual measurements (such as player pose and inbound velocities) that can be extracted from video to support downstream tasks like physical or computational reconstructions. Section 3 covers the methodological details of existing traditional videogrammetry techniques (e.g., point tracking and MBIM) which have been developed and applied for use in HAE research, analysing current capabilities and limitations with regards to measurement accuracy and the wider value that they contribute to the HAE practice and policy discussed in Section 2. Section 4 then explores recent advances in deep learning, with a focus on architectures and applications that offer near-term, actionable improvements to both existing quantitative video-based HAE analysis efforts and developing novel methodologies. Finally, Section 5 reflects on the current state of the field, highlighting key limitations in existing approaches, and outlining future directions for improving the accuracy, scalability, and practical impact of quantitative video analysis of HAEs.

## 2 Context and motivation for video-based quantification

Although the primary focus of this review is to evaluate the current state of video-based methods for quantitatively measuring kinematics associated with HAEs, it is important to situate these techniques within the broader landscape of HAE research. Video-derived measurements, despite current limitations, are used to support downstream applications such as physical reconstruction, computational modelling, and brain injury risk estimation. This section therefore provides a brief overview of these related domains to contextualise the role and potential value which videogrammetric methods add to the wider HAE analysis field, before expanding discussion around specific methodological details in Sections 3,4.

### 2.1 HAE background

The term head acceleration event (HAE) was introduced to address the limitations of the term head impact, which implies direct contact with the head and fails to capture inertial loading from indirect forces (Nguyen et al., 2019; Tierney, 2021). To clarify terminology and support standardisation, the 2022 Consensus Head Acceleration Measurement Practices (CHAMP) group defined a HAE as any event that induces an acceleration response of the head due to short-duration collision forces, applied either directly to the head or indirectly via the body. In contrast, a head impact event (HIE) refers specifically to events involving direct contact with the head (Arbogast et al., 2022).

To structure the field, CHAMP identified six priority areas relevant to HAE research:

1. Study design and statistical analysis (Rowson et al., 2022);
2. Laboratory validation of wearable kinematic devices (Gabler et al., 2022);
3. On-field validation and deployment of wearable kinematic devices (Kuo et al., 2022);
4. Video analysis of HAEs (Arbogast et al., 2022);
5. Physical reconstruction of HAEs (Funk et al., 2022);
6. Computational modelling of HAEs (Ji et al., 2022).

Each of these areas has been addressed by CHAMP through peer-reviewed technical manuscripts, excluding video analysis of HAEs, that is represented in the consensus framework only by a reporting checklist (Arbogast et al., 2022) and an unpublished companion manuscript describing the videogrammetry process (Neale et al., 2022). Despite growing interest in using video to support HAE measurement and interpretation, there remains no review which provides a comprehensive overview of the use of quantitative video-based methods in the literature.

This narrative review addresses that gap by examining how videogrammetric approaches have been used to extract quantitative kinematic parameters from standard video footage, as well as identifying future trends for research in the field. While wearable sensors remain the dominant tool for direct kinematic measurement, video data (particularly from consumer-grade cameras) offers a scalable, complementary avenue for quantifying HAEs. The remainder of this section therefore aims to situate video analysis within the broader HAE ecosystem, highlighting the motivations, benefits, and limitations of its use in both research and applied sport contexts.

### 2.2 Direct measurement

**FIGURE 1:**
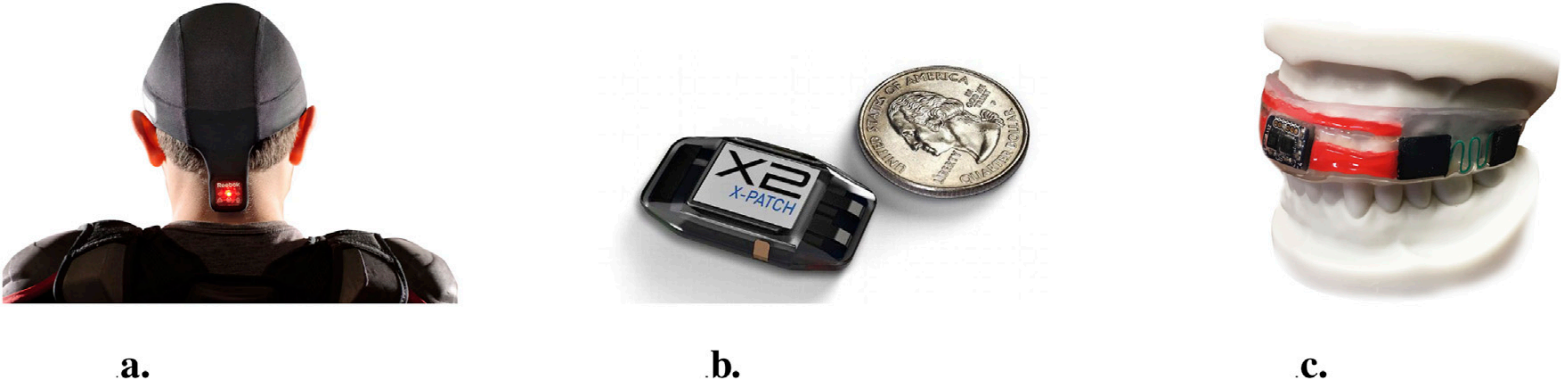
Sensor-based approaches to HAE measurement: **(a)** skull cap (Luna, 2013), **(b)** skin patch (Linendoll, 2013), and **(c)** mouthguard (Wu, 2020).

In the context of this work, direct measurement of HAEs refers to the measurement of kinematic properties (position, velocity, and acceleration) of the head during HAEs. To accomplish this, a range of approaches to measuring head kinematics have been proposed in the literature, from wearable sensors such as headgear, skin patches and mouthguards instrumented with gyroscopes and/or accelerometers (see Figure 1) (Le Flao et al., 2022; Patton, 2016), to videogrammetry approaches where HAE parameters are manually extracted from video footage (Bailey et al., 2018; Gyemi et al., 2023; Stark N. E.-P. et al., 2025; Tierney, 2021).

To detect and record HAEs with wearable sensors, a threshold for linear acceleration (e.g., 10g ) is typically set (Wang T. et al., 2021; King et al., 2016). However, when deployed on-field, such devices often exhibit poor sensitivity, resulting in high false-positive rates (Le Flao et al., 2024). Additionally, a number of these sensor approaches suffer from poor coupling with the skull, leading to noise and undesirable artefacts in the impact kinematic signals (Wu et al., 2016b). Instrumented mouthguards (iMGs) demonstrate superior coupling with the skull (Wu et al., 2016b) but are often considered intrusive and uncomfortable by athletes (Roe et al., 2024; Jones et al., 2022), especially in sports where mouthguard use is not mandatory, such as association football (Kenny et al., 2024). Recent work by Le Flao et al. (2025), Le Flao et al. (2024) has highlighted not only coupling issues, but also the influence of impact location relative to sensor position. Their evaluation of wearable sensors in boxing demonstrated how both sensor type and mounting site can drastically affect signal quality and classification accuracy.

To improve the sensitivity of wearable sensors, many head impact exposure studies also employ manual video analysis to visually confirm detected instances of head impact (Basinas et al., 2022; Patton et al., 2020). However, this is a heavily time-consuming and resource-intensive process. For example, in one study, 163 h of video footage was manually reviewed by a team of 14 raters to verify 217 impact instances (Kuo et al., 2018).

The use of video to qualitatively verify HAE incidence has been well-documented in other reviews (Basinas et al., 2022; Patton et al., 2020), and recent studies have expanded on this by introducing qualitative descriptors into human-rater video analysis frameworks like tackle technique, phase of play, and action of player (Rotundo et al., 2023; Sherwood et al., 2025; Woodward et al., 2025). This review, however, is focused on approaches which can be used to extract quantitative HAE outcome measures directly from video footage using videogrammetric techniques. Point tracking and MBIM methods, for example, have been previously used to extract head kinematics from video footage. A complete overview of these techniques, including specific implementation details and practical considerations, is given in Section 3.

At present, a significant limitation of videogrammetric approaches to direct measurement is that without sufficient camera sampling rates, they are limited to capturing only the pre and post HAE kinematics (Bailey et al., 2018). According to the Nyquist-Shannon theorem, a signal must be sampled at a rate at least twice its highest frequency to avoid aliasing. Wu et al. (2016a) demonstrated that, in unhelmeted sports, gyroscopes with bandwidths of at least 180 Hz (corresponding to sampling rates above 360 Hz ) are necessary to ensure that relative error margins of no more than 10% across various metrics. Therefore, videogrammetric methods like MBIM perform well in controlled laboratory environments with high-sample rate cameras (e.g., 1000 Hz ) (Tierney et al., 2018a), but their application to broadcast video is limited by low frame rates, which violate the Nyquist-Shannon theorem (Edwards et al., 2021; Tierney et al., 2019). Consequently, full video-based direct measurement of HAE kinematics is not yet feasible, and videogrammetric approaches are typically limited to extracting pre- and post-impact velocities, or a reduced subset of HAE parameters used to inform downstream reconstruction and modelling strategies.

### 2.3 Reconstruction and modelling

In the absence of detailed kinematic data for the head during a HAE (as is typical with videogrammetric measurement due to insufficient frame rate/resolution), there are additional downstream computational and physical reconstruction steps which have been utilised alongside quantitative video analysis to gain higher fidelity estimates of head kinematics.

Computational reconstruction approaches typically involve the use of mathematical models of the human body, such as MADYMO (MAthematical DYnamic MOdels) (Bailly et al., 2017; Frechede and McIntosh, 2007; Fréchède and Mcintosh, 2009; Gildea et al., 2024; McIntosh et al., 2014; Tierney and and Simms, 2017; Tierney et al., 2018b; Tierney and Simms, 2019; Tierney et al., 2021), commercially developed finite element models such as the Total Human Model for Safety (THUMS) (Chen et al., 2015; Sharma and Smith, 2024; Yuan et al., 2024b), and custom models developed for specific impact scenarios (Cazzola et al., 2017; Johnson et al., 2016; Perkins et al., 2022). It should be noted that the validation criteria used for these models vary widely, with many human body models originally developed for automotive crash applications, validated using Post Mortem Human Subject (PMHS) experiments, where cadavers of voluntary donors are used as specimens for testing (Wdowicz and Ptak, 2023). Therefore, caution must be exercised when applying these models to sports-related HAEs, in particular where active muscle control has been identified as a significant factor (Tierney and Simms, 2019).

Physical reconstructions, on the other hand, use surrogate models of the human body in laboratory setups, such as crash test dummies or anthropometric test devices (ATDs). These typically include a model of the head instrumented with accelerometers and gyroscopes to measure head kinematics during reconstructed HAEs (Funk et al., 2022). The Hybrid III (Hubbard and McLeod, 1974) and NOCSAE (Hodgson, 1975) headforms have been most commonly used in the literature for physical reconstruction of HAEs. However, the biofidelity of the surrogates used range in sophistication, from headforms rigidly attached to test frames to systems including the head, neck, torso, and limbs with matched joint angles. It has been highlighted that these surrogates may not be able to match an athlete’s pre-impact posture due to their limited head-neck adjustability (Funk et al., 2022), and concerns around unrealistically high stiffness of the Hybrid III neck have also been raised (Gibson et al., 1995), leading to the recent development of more biofidelic sport-specific surrogate necks, for example, (Farmer et al., 2022).

A key consideration in the development of any reconstruction is the choice of HAE parameters used in the reconstruction setup. For computational reconstructions, these parameters are used for simulation setup in the form of initial conditions, such as head and full-body pose, inbound velocities, and impact location and direction (Bailly et al., 2017; Gildea et al., 2024; Yuan et al., 2024a). For physical reconstructions, these parameters are used to set up the test rig, including the initial position of the surrogate headform and the impactor used to recreate the HAE (Funk et al., 2022; Zimmerman et al., 2023). In any case, quantitative video analysis has been identified as a valuable source of data for estimating these parameters, particularly in the context of both computational and physical reconstructions (Funk et al., 2022; Tierney, 2021).

**FIGURE 2:**
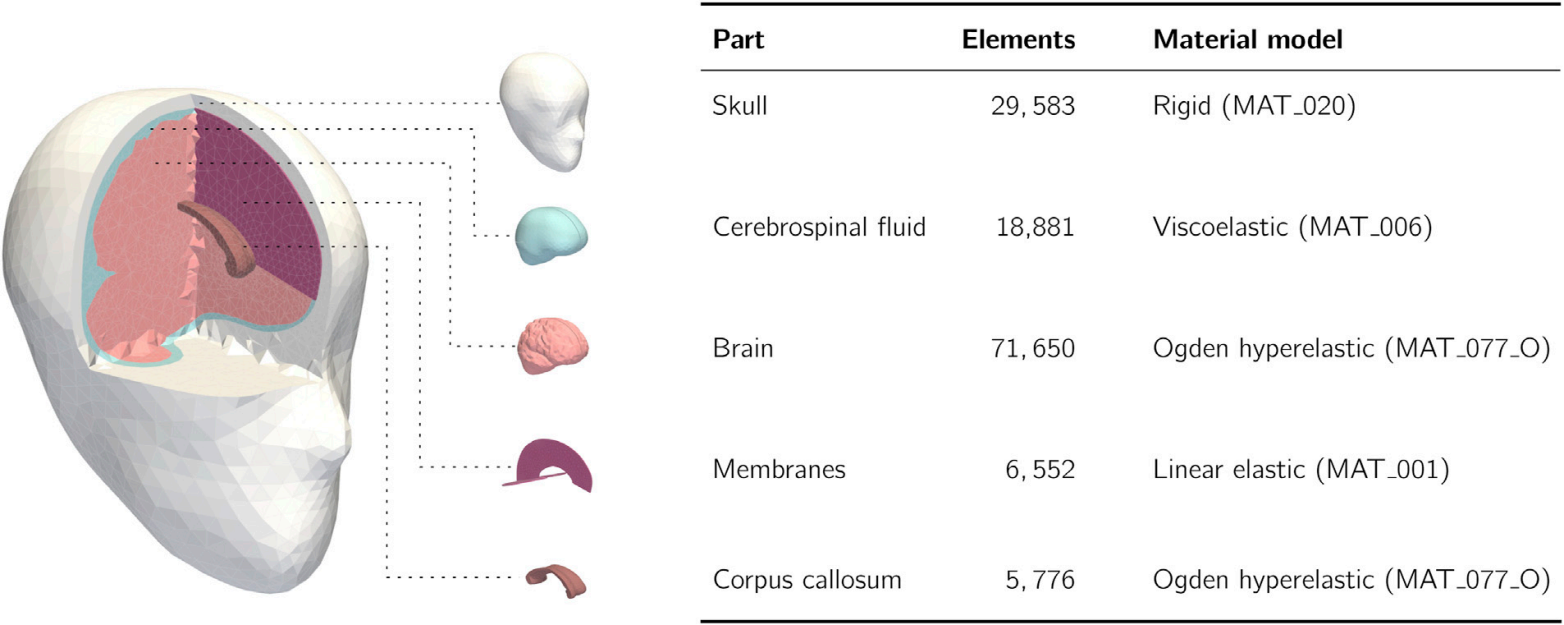
The University of Edinburgh’s Finite Element Head Model (EdiFEHM), with details of its structural components and material models. Adapted from McGill (2022).

Both reconstruction types are used to generate detailed kinematic data for the head, which is often the input to subsequent injury analysis using brain injury criteria (BIC), which provide a link between the kinematic data and risk of brain injury. Simpler models use peak kinematics values (Laksari et al., 2020; Denny-Brown and Russell, 1941; Holbourn, 1943), reduced-order physical models (Gabler et al., 2018; 2019; Laksari et al., 2020; Takahashi and Yanaoka, 2017) and statistical model fitting (Rowson and Duma, 2013; Greenwald et al., 2008), while more advanced approaches use finite element head models (FEHMs), adhering to a range of differing validation criteria (Dixit and Liu, 2017; Madhukar and Ostoja-Starzewski, 2019; McGill et al., 2020) to estimate brain strain, a critical parameter for assessing brain injury risk (Hajiaghamemar et al., 2020; Hajiaghamemar and Margulies, 2021; O’Keeffe et al., 2020; Wu et al., 2020). One such model, The University of Edinburgh’s 50th-percentile male FEHM (EdiFEHM) is depicted in Figure 2.

The peak 95th percentile of maximum principal strain $\left({\text{MPS}}_{95}^{\text{peak}}\right)$ is a commonly used metric (Post et al., 2017; Zhan et al., 2021), although alternative metrics such as regional strain and time-dependent exposure have also been proposed (Perkins et al., 2022; Wu et al., 2020). Despite their detail, FEHMs are also computationally expensive, often requiring hours to simulate a single event. Recent work has explored deep learning for faster strain prediction (Ghazi et al., 2021; Wu et al., 2022), though these methods remain in early stages.

To link video analysis with brain injury modelling, several early-stage workflows have emerged in which pose and velocity estimates derived from video are used to estimate full six degrees of freedom head kinematics which drive brain injury predictions. For instance, Yuan et al. (2024a) proposed a pipeline where human pose (joint angles) extracted from monocular footage is used to initialise a biomechanical human body model simulation, with the resulting head kinematics used as input to a FEHM to estimate brain strain. However, this approach has only been demonstrated in a single case study involving a skiing accident, where predicted high-strain regions were compared to magnetic resonance imaging (MRI) and computed tomography (CT) images from a diagnosed TBI. Commercial interest is also growing, with a recent US patent application from Brainware AI (Karton et al., 2025) describing a system for predicting brain injury directly from video using machine learning techniques, bypassing traditional sensing hardware.

Despite these developments, no workflows that integrate video-derived measurements directly into FEHMs or other brain injury models have yet been validated or deployed at scale. This remains a clear gap in the field, previously limited by the video quality and sampling rate issues discussed previously. To address this, the remainder of this review examines the suitability of current videogrammetric techniques for extracting quantitative head kinematics from video, beginning with traditional methods and their limitations, before turning to emerging deep learning-based approaches and their potential to overcome existing barriers.

## 3 Traditional videogrammetry

### 3.1 Overview

As highlighted by Neale et al. (2022) in the CHAMP report on video analysis of HAEs, video footage can be used to extract both qualitative and quantitative information. Qualitative approaches do not involve precise measurements, but instead rely on visual inspection to identify or confirm HAEs. This can include manually reviewing footage to verify impacts detected by wearable sensors (Kuo et al., 2018; Patton, 2016; Patton et al., 2020), or to classify descriptive features such as tackle type, impact location, and game context (Rotundo et al., 2023; Sherwood et al., 2025; Woodward et al., 2025). By contrast, quantitative approaches involve the extraction of measurable data from video using videogrammetric techniques, such as the positions, orientations, and velocities of the head and other body segments. It is important to distinguish between these two methods, as this review focuses specifically on quantitative video analysis techniques for reconstructing and analysing the mechanics of HAEs. These measurements enable a more detailed biomechanical analysis beyond simple impact verification, and can provide valuable inputs to downstream applications, including physical or computational models for the evaluation of specific HAE case studies.

Photogrammetry is defined as “the science and art of making precise and reliable measurements from images” (Gruen, 1997; Förstner and Wrobel, 2016). Extending this principle to moving images leads to videogrammetry: the science and art of making precise and reliable measurements from video, or still frames extracted from video (Neale et al., 2022; Gruen, 1997). Most quantitative video analysis approaches for HAEs are based on this principle. Traditionally, these techniques can be broadly categorised into manual methods (including point tracking and MBIM) and optoelectronic marker-based tracking methods (Colyer et al., 2018). Modern advances in computer vision and deep learning have also enabled more sophisticated approaches to video-based motion estimation, which build on or replace traditional manual methods. These emerging methods are discussed in detail in Section 4.

In this review, only methods that rely on standard consumer-grade video are considered. This focus reflects the aim to review scalable techniques that can be implemented flexibly in both laboratory and field settings using readily available video cameras, without the need for specialised equipment such as reflective markers, high-end optical tracking systems, or other dedicated hardware. By relying solely on standard video, these methods are adaptable to a wide range of sports contexts and can be applied retrospectively to footage captured during normal play or training, making them practical and widely accessible tools for the study of HAEs.

It is important to note that even with standard video footage there can be significant variation between studies in terms of camera setups, calibration methods, and tracking techniques depending on the context in which a method is developed and applied. This variability can lead to differences in the accuracy and reliability of the measurements obtained, particularly when applying a method outwith the context in which it was developed. For example, a method developed for high-frame-rate laboratory video may not perform well when applied to low-frame-rate broadcast footage (Tierney et al., 2019).

These differences can introduce challenges in comparing results across studies and methods, as well as in interpreting the findings in the context of HAE biomechanics. The CHAMP checklist of information to include when reporting video analysis of HAEs, summarised in Table 1, therefore provides a valuable framework with which methods can be compared and evaluated, and enables complete transparency and reproducibility of methods (Arbogast et al., 2022). While the full checklist is useful for comprehensive reporting of a methodology, including every checklist item in detail for each study considered in this review would add little value. Instead, a streamlined reporting structure is adopted in the comparison tables (e.g., Table 2), which broadly summarise essential elements such as video source, calibration method, outcome measures, and validation approach, so as to support consistent discussion.

**TABLE 1:** Adapted CHAMP checklist for reporting quantitative video reconstruction studies of head acceleration events (Arbogast et al., 2022).

Item	Description
1. Study Design and Case Selection	
1a. Study purpose	State the study aim (e.g., observational, validation)
1b. Video source	Describe where the footage came from (e.g., broadcast)
1c. Eligibility criteria	Define criteria for included events (e.g., sport, impact type)
2. Camera Specifications	
2a. Number/type	Number and type of cameras used
2b. Locations	Camera positions and distances relative to event
2c. Calibration	How cameras were calibrated or aligned
2d. Field of view	Camera lens FOV.
2e. Height	Camera heights above ground
2f. Angle	Camera tilt or orientation
2g. Landmarks	Landmarks used for 3D scaling or scene recreation
2h. Obstructions	How obstructions, glare or lighting were managed
3. Recording Parameters	
3a. Frame rate	Frames per second (FPS); interlaced/deinterlaced
3b. Frame rate variability	Whether variable frame rate was corrected
3c. Shutter speed	Camera shutter speed
3d. Resolution	Video resolution in pixels
3e. Aspect ratio	Display or pixel aspect ratio
3f. Compression	Compression type used
3g. File format	File format(s); any conversions made
4. Video Quality Corrections	
4a. Lens distortion	Methods used to correct lens distortion
4b. Motion blur	How motion blur was handled during tracking
4c. Unstable footage	How unstable video was removed or stabilised
5. Data Processing	
5a. Software	Analysis software used
5b. Resampling	Any data downsampling or frame rate adjustments
5c. Stabilisation	Methods/software for video stabilisation
5d. Filtering	Filters used on kinematic data
5e. Start/end times	How start and end of impact were defined
6. Accuracy and Outcomes	
6a. Accuracy	How measurement accuracy was validated
6b. Outcome measures	What outcomes were calculated and how

**TABLE 2:** Overview of selected point tracking methods used in estimating kinematic outcomes associated with HAEs, including method description and case studies where the method is applied.

Setting	Method	Method description						Case studies
		Camera(s)	Recording Parameters	Calibration	Points Tracked	Outcome Measures	Validation Method	
American Football	Newman et al. (1999)	Multiple stationary and handheld (unspecified number)	60 Hz (deinterlaced)	Field markings	Not specified	Relative impact velocity (2D)	No direct validation, lab re-enactment for plausibility	Newman et al. (2000), Newman et al. (1999); Pellman et al. (2003)
	Campolettano et al. (2018)	1 camera	30 Hz	Field markings (2D grid in Kinovea)	Helmet markers (not specified)	Relative impact velocity (2D)	Timing gates	Campolettano et al. (2018)
	Gyemi et al. (2023)	8 GoPro HERO6 (7 field-level, 1 overhead), various pairs tested	120 Hz, 2704×1520 px, 1/1920s	Field markings and calibration objects	Helmet centre	Helmet-to-ground impact velocity (3D)	Theoretical free fall velocity	Gyemi et al. (2021)
Rugby and/or Australian Rules	McIntosh et al. (2000)	Broadcast match footage	25 Hz	Player anthropometrics; field dimensions	Struck player’s head and striking segment (arm, shoulder)	Closing speed, head velocity change, head impact energy	Timing gates	McIntosh et al. (2000)
	Hendricks et al. (2012)	Broadcast match footage	25 Hz	Field markings	Ball carrier mid-section (hip), tackler upper body	Ball-carrier and tackler velocity and acceleration before contact	Trials with cones at known distances	Hendricks et al. (2012)
Ice Hockey	Post et al. (2018)	5 broadcast cameras	30 Hz, 1920×1080 px	Rink markings (2D grid in Kinovea)	Helmet front edge or helmet-mounted target	Skating speed	High-speed camera (250 Hz)	Post et al. (2019a); Karton et al. (2021); Krbavac et al. (2024); Chen et al. (2023); Butterfield et al. (2023); Michio Clark et al. (2018); Post et al. (2019b); Clark et al. (2018); Post et al. (2021); Kendall et al. (2020); Vale et al. (2022); Meliambro et al. (2022); Kosziwka et al. (2021)
Equestrian	Clark et al. (2020a)	Video sources from governing bodies (cameras unspecified)	23.97–30 Hz	Estimated anthropometrics or known racetrack distances	Unspecified	Head height before falling and body horizontal velocity	Distances compared with known fence and rider heights, no direct velocity validation	Clark et al. (2020a), Clark et al. (2021), Clark et al. (2020b)
Falls in care	Choi et al. (2015)	Fixed surveillance cameras (216 cameras at one site, 48 at another)	30 Hz, 640×480 or 720×480 px	Site-specific calibration grid placed at fall location and in plane of the fall	Pelvis (anterior superior iliac spine), head (ear or forehead) and hand (palm)	Impact velocity, fall duration	3D motion capture (250 Hz)	Choi et al. (2015)
	Shishov et al. (2021)	4 Lorex LNZ44P4B surveillance cameras	30 Hz, 640×480 px	2D grid in plane of fall or 1D participant height	Head, sternum, shoulders, elbows, wrists, pelvis (ASIS), knees, ankles	Vertical and horizontal positions and velocities; angular positions and velocities	3D motion capture (600 Hz)	Shishov et al. (2021)

### 3.2 Camera calibration

In general, the mathematical objective of videogrammetric approaches is to estimate the position and/or orientation of an object in 3D space (or 2D assuming planar motion) based on a combination of 2D image coordinates over time. Traditionally, a crucial precursor to this process is a camera calibration procedure to establish the relationship between 2D image coordinates and 3D world coordinates. The calibration process involves determining the intrinsic (focal length, principal point and distortion coefficients) and extrinsic (position and orientation in the world) parameters of a camera, and can be performed using calibration objects with known geometry or by leveraging fixed known reference geometry in the scene.

Direct Linear Transformation (DLT) is one of the most widely used calibration and reconstruction techniques in biomechanics and sports analysis (Abdel-Aziz et al., 2015; Hedrick, 2008). DLT relates 2D image coordinates to 3D world coordinates via a linear mapping, which can be robustly estimated when a sufficient number of known reference points are visible. Its simplicity, flexibility, and compatibility with both controlled laboratory and field environments have made DLT the backbone of traditional multi-camera calibration workflows in many sports applications.

In single-camera (monocular) setups where it is reasonable to assume that all tracked motion occurs on a known planar surface (e.g., a football pitch or ice rink), 2D-DLT is generally used (Hedrick, 2008) (although more complex non-linear techniques also exist to address issues such as radial lens distortions (Zhang, 1999; Dunn et al., 2012)). 2D-DLT establishes a projective mapping between 2D world coordinates (x,y) on the plane and 2D image coordinates (u,v) using a homography, as described by Equation 1:

$$\left[\begin{array}{c} u\\ v\\ 1 \end{array}\right]\propto H\left[\begin{array}{c} x\\ y\\ 1 \end{array}\right]$$ (1)

Here, H is a 3×3 homography matrix with 8 degrees of freedom, estimated using at least four non-collinear point correspondences between the scene and the image. Calibration points can often be extracted from visible field markings of known dimensions (e.g., penalty spots, sidelines, hash marks), allowing accurate determination of an object’s planar displacement and velocity (Post et al., 2018; Tierney et al., 2019; Gyemi et al., 2023).

When full 3D motion tracking is required (such as capturing a helmet’s movement through space to estimate the velocity of a fall) 3D DLT must be used. This requires synchronised views from at least two calibrated cameras with overlapping fields of view. A set of known 3D reference points (x,y,z) is then used to establish the projection parameters of each camera using Equation 2:

$$\left[\begin{array}{c} u\\ v\\ 1 \end{array}\right]\propto P\left[\begin{array}{c} x\\ y\\ z\\ 1 \end{array}\right]$$ (2)

**FIGURE 3:**
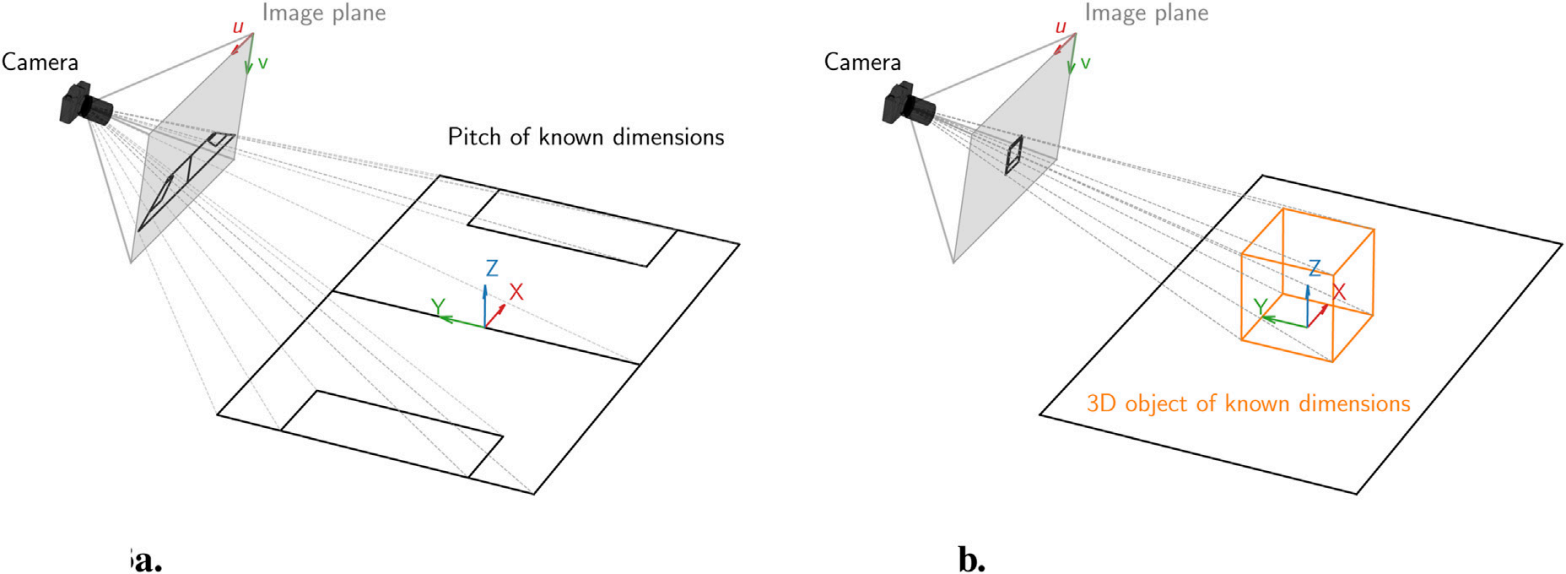
Illustration of the camera calibration process using **(a)** known reference points in the scene, such as field markings for 2D planar calibration, and **(b)** a typical 3D calibration object, such as a chessboard or calibration grid, for 3D calibration.

The matrix P is the 3×4 camera projection matrix containing both intrinsic and extrinsic parameters. It can be estimated using at least six non-coplanar 3D–2D correspondences. Calibration is typically performed using a known 3D object (e.g., a checkerboard or a wand moved throughout the scene) or by leveraging fixed known 3D geometry. Figure 3 illustrates the difference between 2D and 3D calibration workflows.

In both 2D and 3D calibration contexts, the placement of cameras plays a critical role in reconstruction accuracy. For 3D calibration in which a multi-camera setup is used, achieving a wide angular separation is important to ensure strong triangulation geometry and reduce depth ambiguity (Hartley and Zisserman, 2004). Cameras should ideally capture the scene from multiple angles with overlapping fields of view, and their positions should be stable and known throughout the recording. In 2D calibrations, for example, using known field markings, the camera must capture a sufficient portion of the calibrated surface (e.g., a flat pitch) without extreme perspective distortion (Szeliski, 2022). Oblique or high-angle views can reduce the reliability of 2D-to-3D mappings, especially when estimating motion in depth.

In scenarios where the camera is not stationary (as is often the case for “in-the-wild” sports video or handheld footage) DLT calibration can be performed independently for each frame, provided that enough reliable 2D–3D correspondences are visible. This results in a time-varying projection matrix ( P(t) or H(t) ) that captures the frame-specific pose of the camera. Other approaches such as camera stabilisation techniques (Bailey et al., 2018) and structure-from-motion (SfM) can also be used to estimate time-varying camera geometry directly from natural scene features (Schonberger and Frahm, 2016).

### 3.3 Point tracking

Once cameras have been calibrated, points of interest (such as anatomical landmarks, joint centres or the estimated centre of mass of the head) can be identified and tracked across successive video frames. In general, point tracking methods involve the identification and subsequent tracking of specific points or landmarks on an athlete’s body or equipment over a sequence of images. Historically, this process was performed through “manual digitisation” or “point-click” techniques, whereby researchers meticulously clicked on and marked points of interest frame-by-frame (Colyer et al., 2018; Yeadon and Challis, 1994; Hedrick, 2008). While advancements have introduced semi-automated tracking algorithms, these often still require initial manual seeding to define the points of interest (Hedrick, 2008). Recent developments in computer vision and deep learning have also enabled fully automated markerless tracking systems, which will be discussed in more detail in Section 4.

In the context of HAEs, accurate point tracking has primarily been used to estimate impact velocities and other collision parameters, which in turn inform physical reconstructions, computational simulations, and injury risk assessments. Some of the earliest attempts at estimating head accelerations in sporting collisions relied on point tracking techniques in the absence of wearable sensors to measure player speeds around the instant of impact, which were then used in physical reconstructions to estimate head kinematics (McIntosh et al., 2000; Newman et al., 1999; Withnall et al., 2005).

**FIGURE 4:**
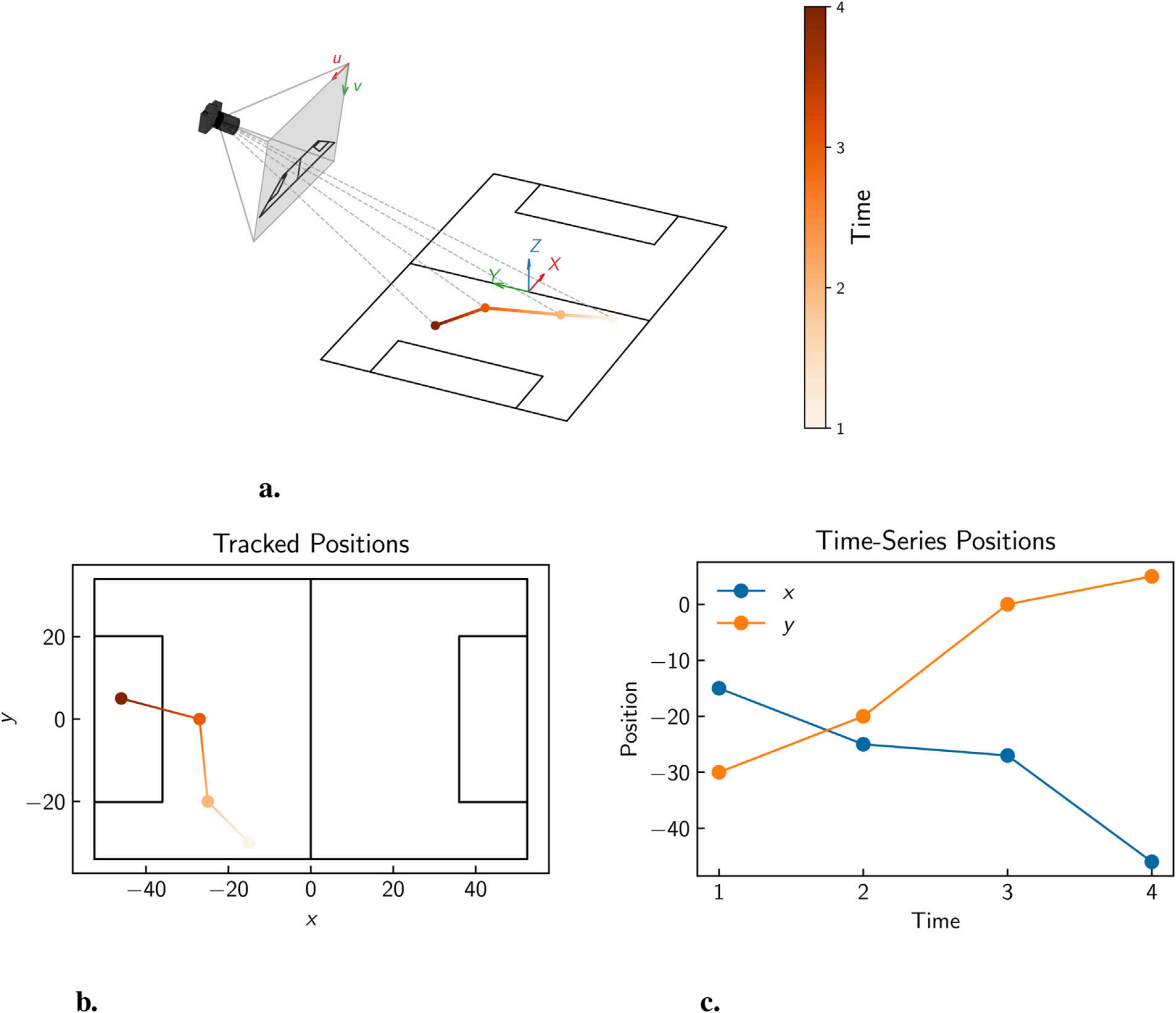
Illustration of 2D point tracking, showing **(a)** the movement of a point in 3D space over time, viewed by a single calibrated camera, **(b)** the tracked x and y positions (assuming planar motion), and **(c)** the corresponding time series data for the tracked position.

**FIGURE 5:**
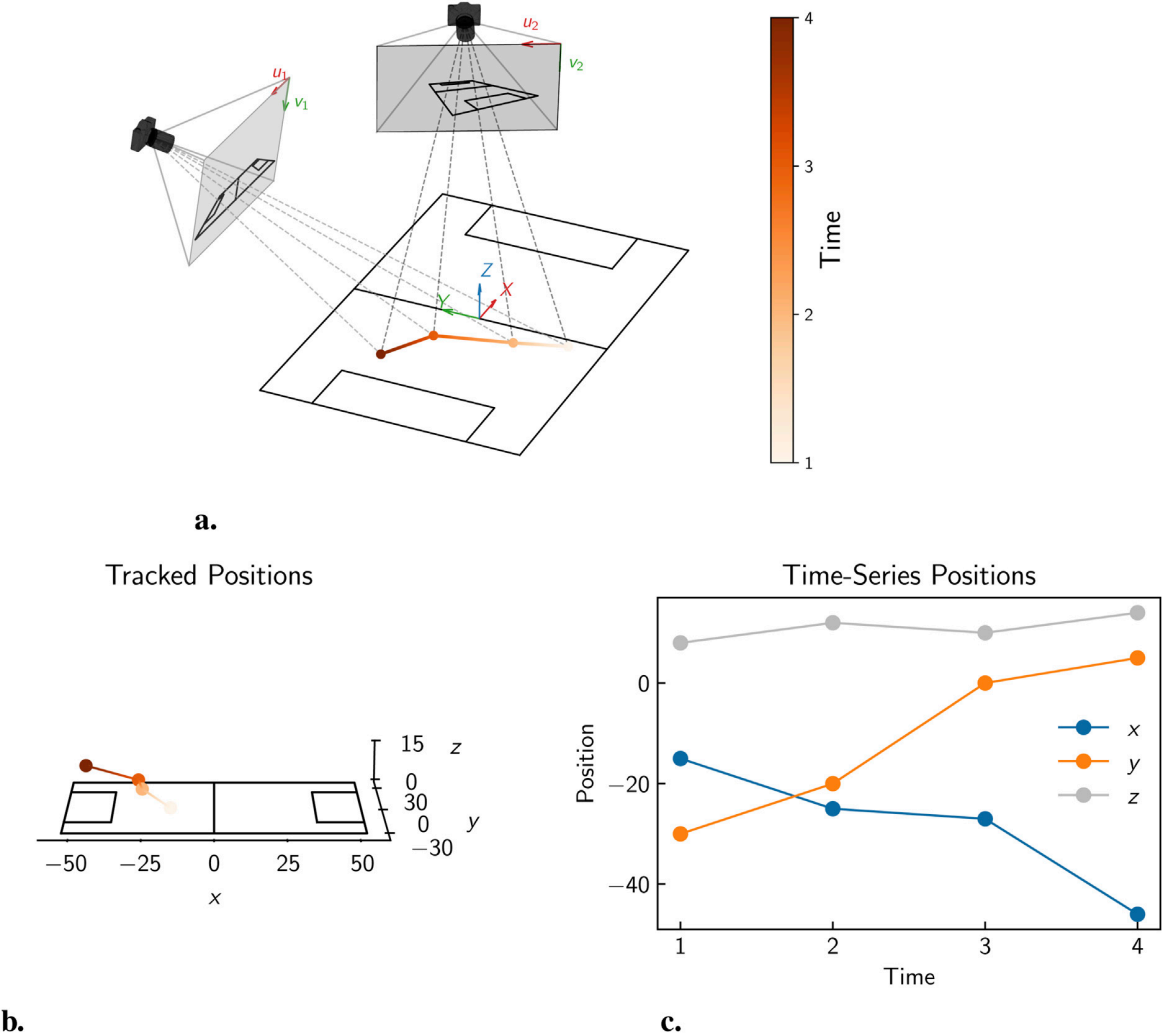
Illustration of 3D point tracking, showing **(a)** the movement of a point in 3D space over time, viewed by two calibrated cameras, **(b)** the reconstructed x , y , and z positions, and **(c)** the corresponding time series data for the tracked position.

A range of both 2D and 3D point tracking methods have been developed in the literature, with the choice of method often depending on the available camera setups in a chosen setting and the specific requirements of the analysis. In 2D point tracking, a single calibrated camera is used to track points in a plane, while in 3D point tracking, two or more calibrated cameras are used to reconstruct the 3D position of points in space. A schematic illustration of the process of 2D point tracking using a single camera is shown in Figure 4. In this example, a point in 3D space is viewed by a single calibrated camera, and the tracked x and y positions are extracted as time series data. Similarly, Figure 5 illustrates 3D point tracking, where a point in 3D space is viewed by two or more calibrated cameras, allowing the tracked x , y , and z positions to be reconstructed as time series data.

Table 2 provides a summary of a number of point tracking methods which have been used in the literature to estimate kinematic outcome measures associated with HAEs. Where specified in the original studies, the table includes details of the setting and method specifications (including camera configurations, recording specifications, calibration approaches, points tracked, kinematic outcomes, and validation methods) in order to provide context for each of the methods in a manner consistent with the CHAMP reporting checklist in Table 1. Table 2 also includes references to relevant case studies demonstrating practical applications of these methods in real-world scenarios.

Note that when a camera’s sample rate is referred to as deinterlaced, this means that the original interlaced video (typical with broadcast footage) has been converted to a progressive format, effectively doubling the frame rate for analysis purposes. When measuring positions in interlaced frames, there are two possible positions, whereas the deinterlaced frames correctly provide both positions in sequence, thereby improving the accuracy of motion tracking and kinematic calculations. However, the accuracy of deinterlaced footage depends on the method used: for instance, simple line-doubling or field-repeating methods may introduce artefacts, while motion-compensated deinterlacing offers more accurate reconstruction at the cost of computational complexity (Sugiyama and Nakamura, 1999). As such, appropriate deinterlacing techniques should be applied and clearly reported when analysing interlaced footage in HAE studies (Neale et al., 2022).

As shown in Table 2, a wide variety of camera configurations, calibration procedures, tracking strategies, and outcome measures have led to the development of a diverse range of point tracking methods for analysing HAEs. These methods have supported a range of practical applications, influencing both research and policy. In some cases (such as studies in American football, rugby, and Australian Rules) the estimated velocities from point tracking have been used primarily as surrogate measures of impact magnitude, without being applied in further modelling or reconstruction. For example, Campolettano et al. (2018) noted that their method, alongside wearable sensors, could be directly used to inform the design of impact testing conditions that better reflect youth football collisions.

In contrast, other studies have further leveraged point tracking as a foundation for more in-depth biomechanical analysis. The approach developed by Post et al. (2018), which involved tracking helmet markers to estimate skating speed in ice hockey, has been widely applied in follow-up HAE research. Extracted velocities from this method have been used to drive physical reconstructions investigating the influence of athlete age (Chen et al., 2023), playing position (Butterfield et al., 2023), and rule changes related to body contact (Krbavac et al., 2024), to name a few examples only. Crucially, these reconstructions have enabled further evaluation of brain injury risk based on the resulting head kinematics, demonstrating that a relatively simple point tracking approach can provide the foundation for sophisticated biomechanical modelling and injury risk assessment.

However, it should be emphasised that each method has been designed and validated within the context of a specific sport or activity and thus may not be directly transferable to other settings without additional validation. For example, several studies in American football (Taylor et al., 2019; Karton et al., 2020; Vale et al., 2022; Meliambro et al., 2022) have cited the accuracy demonstrated by Post et al. (2018) for a 2D point tracking method for measuring player impact velocities in ice hockey to support their own measurement approaches. However, differences in camera configurations, calibration protocols, and the features tracked mean that such validation results may not be generalisable beyond their original context. This highlights the importance of initiatives such as the CHAMP project, which advocates for transparent reporting and rigorous validation of video-based analysis methods for HAEs (Arbogast et al., 2022).

The observed differences in camera setups across point tracking studies also have important implications for the scalability and wider applicability of these methods. In particular, the financial and logistical demands associated with high-end camera configurations can limit their use beyond elite sporting contexts. For example, studies by McIntosh et al. (2000) and Hendricks et al. (2012), conducted in rugby and Australian Rules football, used footage from multiple broadcast-quality cameras to reconstruct player kinematics. While such setups were shown to yield high-fidelity data, and may be readily available in elite environments where broadcast footage is routinely captured, they are clearly less suited to large-scale research efforts focused on non-professional settings, where such infrastructure is unlikely to exist. By contrast, lower-cost approaches such as the single pitchside camera method used by Campolettano et al. (2018), or the multi-GoPro configuration employed by Gyemi et al. (2021), offer more practical and affordable alternatives. These methods can be deployed more flexibly in training sessions or amateur competitions with minimal setup, reducing both financial barriers and the practical burden of collecting and processing large volumes of video data.

This variety in methods and their practical applications has also contributed to a range of reported accuracies. When measuring the velocities of ice skaters during slow and fast trials, Post et al. (2018) observed variations in the accuracy of their 2D point tracking method, with mean absolute errors of approximately 0.2–0.7 m/s for slow skating speeds (around 4.5 m/s) and 0.4–1.3 m/s for fast speeds (around 7 m/s). Hendricks et al. (2012) reported mean differences of 0.11–0.62 m/s between their video-derived speeds and timing gates on rugby fields, and in American football, Campolettano et al. (2018) measured player velocities ranging from 0.2 to 5.4 m/s, reporting mean errors under 10% (suggesting absolute errors up to about 0.5 m/s). Using a 3D point tracking method with multiple cameras, Gyemi et al. (2023) validated helmet impact velocities in controlled drop tests (4.5–6.1 m/s) and found relative errors below 3.4% in optimal camera configurations, equivalent to absolute errors below 0.22 m/s, but noted higher errors (up to 10.9% or 0.55 m/s) when camera angles were suboptimal. However, inconsistencies in validation procedures, sample sizes, and the choice of reference standards make it difficult to directly compare the accuracy of different methods. Standardised reporting of camera placements, calibration steps, and statistical validation metrics will support clearer cross-study comparisons, but direct comparison will remain challenging due to the inherent variability in sports contexts, camera setups, and tracking techniques.

In addition, few studies have explicitly quantified the manual workload associated with these approaches. Nevertheless, it is evident that methods requiring manual digitisation or semi-automated tracking with manual point seeding are inherently limited in their scalability, particularly for exposure studies that demand the analysis of hundreds or thousands of HAEs across many frames. For example, Campolettano et al. (2018) analysed only a subset (50) of the 336 high-acceleration game impacts recorded, citing practical limitations such as insufficient field markings or camera movement preventing the establishment of a reliable reference grid. These constraints highlight how manual or labour-intensive methods, while feasible for focused case studies, can become prohibitively time-consuming in larger-scale applications.

Finally, another crucial limitation of most point tracking methods is that they are primarily used to estimate translational parameters, such as linear velocity or displacement, while providing little or no information about rigid body orientations, such as that of the head, for example,. This is because tracking a single point on an object does not sufficiently constrain its rotation in three-dimensional space. Therefore, multiple (3 or more) non-collinear markers are required to resolve rotational degrees of freedom reliably in this context (Marchand et al., 2016), which comes with the challenges of marker visibility and extensive manual effort. As a result, point-tracking methods typically do not capture the full six degrees of freedom motion of the head during HAEs. This is a significant drawback, since rotational motion has been shown to be a major contributor to brain injury risk (Ji et al., 2014; Bian and Mao, 2020; Kleiven, 2013). Consequently, alternative approaches have been developed in an effort to estimate both translation and rotation parameters for HAEs.

### 3.4 Model-based image matching

**FIGURE 6:**
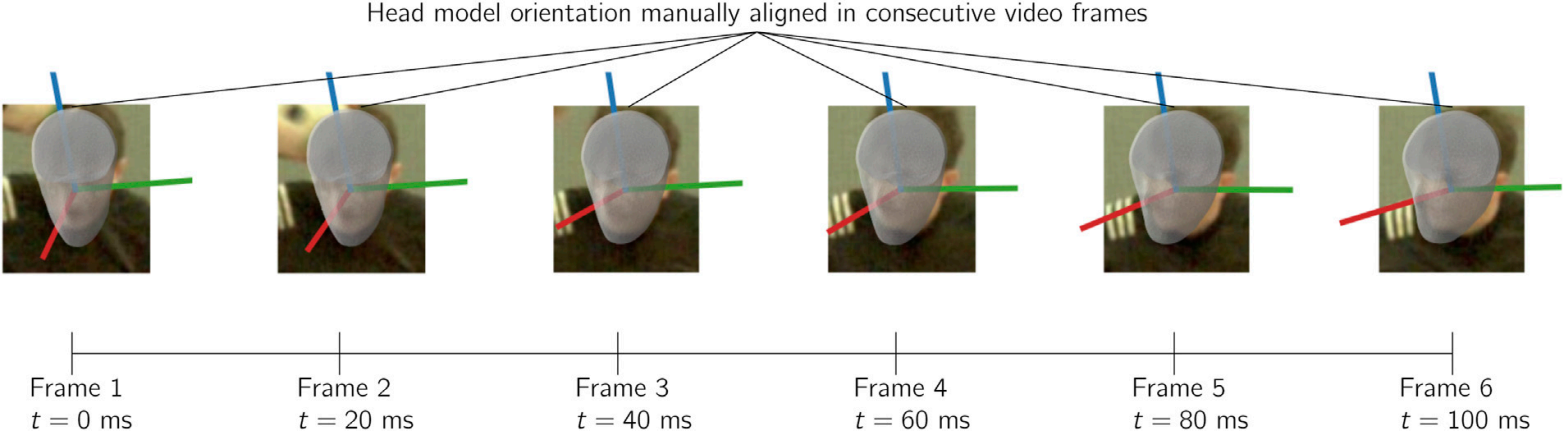
Overview of the MBIM approach to HAE measurement for a video sampled at 50 Hz.

An alternative to point tracking methods is the use of model-based image matching (MBIM) techniques, which involve aligning a 3D model of an object of interest (e.g., a human body or helmet) with the corresponding observed instance of the object in the video frames. Originally developed and validated to measure 6 DOF motion for the pelvis, hip and knee during simple movements such as jogging and side step cutting in a laboratory setting (Krosshaug and Bahr, 2005), MBIM methods have since been adapted to estimate kinematic parameters associated with HAEs in a variety of sports contexts. Figure 6 illustrates an example in which a 3D head model has been aligned with the observed head in frames of a video depicting a head impact.

Several studies cite the use of “uncalibrated” video as a benefit of MBIM methods (Stark N. E.-P. et al., 2025; Krosshaug and Bahr, 2005). While these studies do not use explicit camera calibration prior to video recording, as with a number of the point tracking methods in the previous section, they utilise objects of known dimensions present in the scene to establish the necessary scale and spatial references needed for meaningful measurement. For example, some studies achieve this by using known field markings (Tierney et al., 2018a) or full 3D scans of stadiums (Bailey et al., 2018; Jadischke et al., 2019) to reconstruct camera positions and orientations relative to a global coordinate system, enabling measurement of both absolute position and orientation of the head or helmet in a global frame. Stark N. E.-P. et al. (2025), bypass the need for environmental calibration altogether by relying on a known size 3D headform or helmet model being matched directly to the video. This model calibration approach defines a local scale around the head itself, which enables estimates of head position, depth motion, and rotational pose relative to the camera to be obtained, even in footage where the wider scene remains unmeasured. Therefore, while MBIM methods may not require explicit camera calibration in the traditional sense, the fundamental requirement of a reference object or model of known size remains essential for accurate measurement.

Table 3 summarises a number of MBIM methods which have been used in the literature to estimate kinematic outcome measures associated with HAEs. As with Table 2, the table includes details of the setting and method specifications in a similar manner to the CHAMP reporting checklist in Table 1.

**TABLE 3:** Overview of selected model-based image matching methods used in estimating kinematic outcomes associated with HAEs, including method description and case studies where the method is applied.

Setting	Method	Method description						Case studies
		Camera(s)	Recording Parameters	Calibration	Bodies Tracked	Outcome Measures	Validation	
American Football	Bailey et al. (2018)	11 broadcast cameras in seven standard recording locations	60 and 240 Hz (deinterlaced); 1,000–18,600 px per helmet	3D laser scan of field and stadium	Helmet	Linear and rotational velocities; pre, during and post HAE	3D motion capture (1000 Hz)	Bailey et al. (2018), Bailey et al. (2020)
	Jadischke et al. (2019)	15 stationary GoPro Hero6 action cameras	Lab validation: 240 Hz, 1920×1080 px; On-field: 120 Hz, 2704×1520 px	3D laser scan of field and stadium	Head (skull segment)	Linear and rotational velocity; pre-impact and resultant change	Anthropomorphic Test Dummy (ATD) sensor data	Jadischke et al. (2019), Jadischke et al. (2020)
Skiing	Yamazaki et al. (2015)	4 cameras, unspecified location/motion	50 Hz (deinterlaced); 1920×1080 px	Terrain coordinates and camera positions measured with global navigation satellite system	5-segment skeletal model: pelvis (parent segment), abdomen, chest, neck, head	Linear (normal and tangential to slope) and rotational (frontal plane tilt) pre- and post-impact velocities; impact angle	No direct validation, uncertainty estimated using constant velocity assumption	Yamazaki et al. (2015)
Falls in care	Stark et al. (2025b)	1 stationary camera, various heights and distances	30 Hz; 1920×1080 px	Model-based calibration (to-scale 3D headform and helmet models)	Headform in drop tests; helmet/head in participant ladder falls	Linear impact velocities	Speed gate (drop tests), 3D motion capture (ladder falls)	Stark et al. (2025b), Stark et al. (2025a)
Automotive	Tierney et al. (2018a)	3 stationary digital video cameras	100 Hz; 800×600 px	Known laboratory dimensions	Head (skull segment)	Linear and rotational velocities of head pre, post and during impact	3D motion capture (1000 Hz)	Tierney et al. (2018a)
Rugby	Tierney et al. (2019)	3 broadcast cameras	25 Hz; 1280×720 px	Field dimensions	Head and pelvis	Linear and angular velocities (maximum change)	No direct validation	Tierney et al. (2019)

As shown in Table 3, there is once again considerable variation in camera setups used across MBIM studies. These range from multi-camera broadcast systems (Bailey et al., 2018), to pitch-side GoPro arrays (Jadischke et al., 2019), and even single-camera surveillance footage (Stark N. E.-P. et al., 2025). As noted earlier, calibration procedures also differ greatly in both cost and practicality. At one end of the spectrum are resource-intensive approaches involving full 3D scans of stadium environments (Bailey et al., 2018; Jadischke et al., 2019), while other studies used more accessible methods involving localised calibration by aligning a 3D model of the headform or helmet directly to the video frames (Stark N. E.-P. et al., 2025).

These differences extend beyond calibration to the broader implementation of MBIM, particularly in how six degree of freedom model alignment, and subsequent calculation of kinematic data, is achieved. Studies have used a range of software environments, including commercial packages such as Poser (Tierney et al., 2018a) and SideFX Houdini (Bailey et al., 2018), as well as in-house tools developed using the Godot game engine (Stark N. E.-P. et al., 2025). The model positioning methodology also varies. For instance, Bailey et al. (2018) describe a two-stage process in which the translational path of an American football helmet is first estimated by fitting an outline to the helmet in each frame, followed by manual adjustment of all six degrees of freedom to align the virtual and video helmet markings. Other studies describe approaches such as self-assessing alignment based on minimising discrepancies in visible facial features (e.g., edges, nose, eyes, mouth) (Stark N. E.-P. et al., 2025), or manipulating a 3D skeleton model frame-by-frame to match the skull orientation to that of the cadaver model present in the video (Tierney et al., 2018a). Once model alignment is complete, positions and orientations are typically converted into velocities using numerical differentiation. To mitigate the noise introduced by this numerical differentiation process, various strategies have been employed, ranging from fitting interpolating cubic spline polynomials (Tierney et al., 2018a) to applying traditional filtering techniques, such as low-pass Butterworth filters guided by fast Fourier transforms of the positional data (Bailey et al., 2018).

Naturally, it follows that the variety in setups associated with MBIM methods has led to a range of reported accuracies. Using multiple camera views and environmental alignment, Tierney et al. (2018a) reported errors ranging from 0.42±0.07 m/s to 1.29±0.21 m/s for head velocities spanning −10.9 to 1.2 m/s. In American football, Bailey et al. (2018) and Jadischke et al. (2019) applied MBIM with 3D scans of the playing environment to calibrate camera positions, achieving mean errors of about 9% (0.4 m/s) and 10.7% (0.24 m/s), respectively. More recently, Stark N. E.-P. et al. (2025) demonstrated that a model-based calibration can produce a comparable error of approximately 0.7±9.5% ( 0.01±0.33 m/s), for resultant head impact speed in uncalibrated single-camera videos.

Despite methodological differences between MBIM and point tracking techniques, the outcome measures reported in MBIM studies are often similar to those derived from point tracking (primarily translational kinematics) with the notable addition of rotational velocity estimates in some cases. As with point tracking studies, the extracted velocity data are rarely used beyond basic comparisons of raw magnitudes. For instance, Bailey et al. (2020) used MBIM to compare impact velocities across different contact scenarios (e.g., helmet-to-helmet, helmet-to-shoulder, etc.) with the goal of producing a biomechanical characterisation of concussive events, but did not extend their analysis into further modelling or simulation.

Of the studies listed in Table 3, only Stark N. E. P. et al. (2025) used MBIM-derived velocities as input for downstream modelling beyond simple magnitude comparisons. In their study of elderly falls, linear head impact velocities obtained from MBIM were used to inform drop-weight impact testing conditions. Outside the peer-reviewed literature, England (2025) also applied a MBIM approach to extract helmet orientation at the moment of impact between ball and helmet in cricket, which was then used to support physical reconstructions of injury events. To the authors’ knowledge, however, no published computational modelling studies of HAEs have explicitly detailed the use of MBIM-derived kinematics to initialise their simulations, despite the clear potential of MBIM to provide full-body pose data for this purpose (Bailly et al., 2017; Frechede and McIntosh, 2007; Fréchède and Mcintosh, 2009; Gildea et al., 2024; McIntosh et al., 2014; Tierney and and Simms, 2017; Tierney et al., 2018b; Tierney and Simms, 2019; Tierney et al., 2021). For example, Fréchède and Mcintosh (2009) describe using HYPERMESH to manually recreate pre-impact player positions for multibody simulation in MADYMO (a method later referenced by McIntosh et al. (2014)) but do not provide methodological detail or validation of a complete MBIM protocol. In other cases, researchers have opted for alternative approaches: Tierney and Simms (2019) used marker-based motion capture to stage rugby union tackles in the lab, from which initial conditions for MADYMO simulations were extracted, while Gildea et al. (2024) employed a deep learning-based pose estimation method (see Section4) to initialise similar computational reconstructions of cyclist falls.

A significant drawback of MBIM, perhaps limiting its widespread use for informing model positioning for computational and physical reconstructions, is the time-consuming nature of the manual model alignment process. For example, a case study of a single rugby HAE required an estimated 60 h of manual effort by one researcher to complete the MBIM procedure across three camera views, where only the orientation of the head was estimated in each frame (Tierney et al., 2019). When full-body orientation is required, this effort would increase substantially. Results are also sensitive to small inconsistencies in how the model is positioned and scaled in each frame, which Stark N. E.-P. et al. (2025) addressed by using an iterative re-tracking process to refine alignment until the measurement error fell below a set threshold, emphasising the need for well-trained operators. This repeated refinement further adds to the time demands, limiting the practicality of MBIM for large datasets or real-time applications.

## 4 Deep learning

As discussed earlier, videogrammetric analysis of HAEs has often been limited by practical and technical limitations such as occlusions, frame rate restrictions, highly labour intensive processes, and the difficulty of recovering six degrees of freedom motion from uncalibrated monocular footage. In recent years, advances in machine learning (particularly deep learning) have provided powerful solutions to these challenges in other domains, enabling scalable and automated extraction of complex spatiotemporal features from video data. These methods are now gaining traction in the context of HAE analysis, and more specifically in quantitative video analysis, where they hold significant promise for reducing manual workload and enhancing the accuracy and completeness of motion estimation.

Traditional ML methods (such as support vector machines and random forests) have been effective in processing lower-dimensional wearable sensor data. However, these methods do not scale effectively to the high-dimensional, non-linear nature of video data. The shift to deep learning has therefore not only improved performance but also enabled new applications, particularly in video-based pose estimation and motion reconstruction.

This section traces the evolution of machine learning techniques relevant to the quantitative analysis of HAEs, beginning with early sensor-based applications of classical methods and progressing toward recent advances in deep learning for image- and video-based approaches. It also draws on developments from related domains that demonstrate promising potential for advancing HAE quantification specifically through video analysis.

### 4.1 Context and motivation

Traditional machine learning methods have played a foundational role in classifying, detecting, and quantifying HAEs from wearable sensor data. These approaches typically involve hand-engineered features derived from time-series signals such as linear acceleration and angular velocity. For example, Wu et al. (2017) used a support vector machine to detect impacts from accelerometer data, while Gabler et al. (2020) expanded this work by benchmarking multiple classification algorithms and handcrafted feature sets. Zhan et al. (2023) applied random forests to subtype impacts and used nearest-neighbour regression to estimate brain strain, showing the potential of classical ML models in injury modelling. Other applications include distinguishing between head and body impacts (Goodin et al., 2021) and generating synthetic head impact signals using low-rank representations via principal component analysis (PCA) (Arrué et al., 2020).

More relevant to quantitative video analysis are the earliest algorithmic attempts to automate the estimation of human and object motion from video. Silhouette-based techniques such as the visual hull method (Laurentini, 1994) formed the foundation for early markerless motion capture systems (Corazza et al., 2010), enabling coarse reconstruction of human movement without physical markers. As machine learning methods developed, classical algorithms were adopted for pose and motion estimation. These included the use of random decision forests for real-time 3D human pose estimation from depth images (Shotton et al., 2011), support vector machines paired with handcrafted spatiotemporal features for action recognition (Wang et al., 2011), and kernel ridge regression and nearest-neighbour approaches for estimating full-body 3D pose from monocular video (Ionescu et al., 2014). However, these early methods were typically constrained to laboratory environments with controlled lighting and camera setups. In the context of HAEs, practical limitations, such as the scarcity of ground truth data in real-world sport settings and the visual challenges posed by occlusions and fast motion (to which early methods were not robust), have limited their widespread adoption for quantitative video analysis in this field.

**FIGURE 7:**
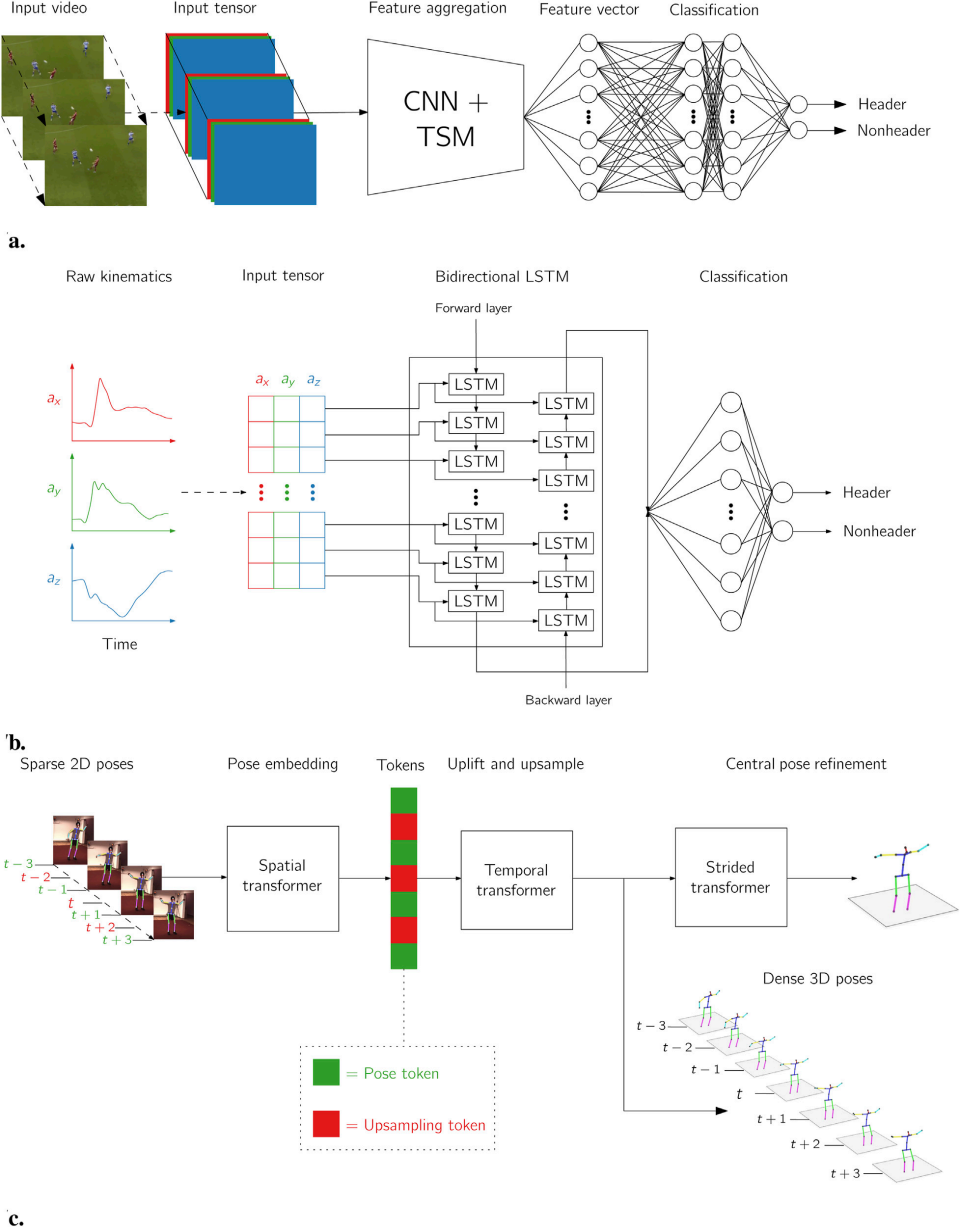
Examples of deep learning architectures applied to video analysis of HAEs and pose estimation domains: **(a)** a convolutional neural network (CNN) architecture for header classification (Rezaei and Wu, 2022), **(b)** a recurrent neural network (RNN) architecture for header detection (Kern et al., 2022), and **(c)** a transformer architecture for temporal upsampling (Einfalt et al., 2023).

The advent of deep learning (LeCun et al., 2015) has driven rapid advancements in video analysis, significantly expanding what is possible when estimating human movement from “in-the-wild” images (Bogo et al., 2016). Convolutional Neural Networks (CNNs) have emerged as the foundational architecture for spatial feature extraction in images and video. Introduced by Lecun et al. (1998) and popularised in computer vision tasks by Krizhevsky et al. (2012), CNNs can learn spatial filters that capture local patterns such as edges, shapes, and textures. In the context of HAE analysis, Rezaei and Wu (2022) used a CNN backbone to classify football headers from cropped video clips. To capture temporal dependencies, Recurrent Neural Networks (RNNs) and particularly LSTMs (Hochreiter and Schmidhuber, 1997) have been widely used. These models are capable of modelling sequential data by maintaining hidden states that evolve over time, making them well-suited to recognising events that unfold across multiple timesteps or frames. Kern et al. (2022) employed LSTM networks to detect HAEs in time-series data from wearable sensors. More recently, transformer architectures (Vaswani et al., 2017) have gained popularity due to their scalability and ability to model long-range dependencies using self-attention mechanisms. These models have seen early applications in biomechanics, including pose tracking, video frame interpolation, and temporal upsampling. Einfalt et al. (2023), for instance, used a transformer-based model to uplift and upsample low sample rate 2D pose estimations to higher temporal resolutions in 3D. Figure 7 illustrates the representative examples of CNN, LSTM, and transformer architectures mentioned above.

With modern deep learning methods demonstrating strong performance in human pose estimation, and video-based motion analysis more broadly, their application to HAEs is beginning to emerge. The remainder of this section highlights these early deep learning efforts, both those applied directly to HAE analysis and promising approaches from adjacent domains that offer clear potential for future adaptation.

### 4.2 Action detection

While this review focuses primarily on methods for the quantitative measurement of HAEs from video, it is important to acknowledge the foundational role that automated event detection and localisation plays in enabling such measurements at scale. In large video datasets, the initial task of identifying where and when HAEs occur is often the most time-consuming and labour-intensive stage of the analysis pipeline (as described in Section 2.2). Manual review workflows, such as those employed in many exposure studies, involve frame-by-frame inspection of footage to locate potential HAEs, a process that is not only slow and resource-intensive but also prone to inconsistency across annotators (Patton et al., 2020). Therefore, although these methods may not yield quantitative kinematic data directly, their ability to efficiently localise events of interest is critical for making subsequent measurement methods (e.g., point tracking, MBIM, or pose estimation) practically viable.

The detection of HAEs by applying deep learning techniques to wearable sensor data has been considered. Motiwale et al. (2016) explored the use of multilayer perceptrons for impact detection, while Kern et al. (2022) introduced long short-term memory (LSTM) networks to account for temporal dynamics. Raymond et al. (2022) developed a physics-informed neural network that integrates domain knowledge with data-driven learning. More recent works have extended deep learning applications to tasks such as kinematic denoising (Zhan et al., 2024a) and the estimation of impact characteristics (e.g., location, speed, force) from time-series sensor data using LSTM-based models (Zhan et al., 2024b).

With respect to video analysis, specifically, Giancola et al. (2023) and Rezaei and Wu (2022) fine-tuned existing CNN-based models to automatically detect headers in football, while Azadi et al. (2024) addressed a similar problem regarding impacts in ice hockey. Mohan et al. (2025) extended this line of work to rugby, using temporal action localisation techniques to detect head-on-head collisions. Reported model accuracies vary depending on sport and dataset complexity, as well as the evaluation metrics used to benchmark model performance. For example, Rezaei and Wu (2022) reported a sensitivity of 92.9% (indicating the model’s ability to correctly identify true head contact events) and a precision of 21.1%, reflecting a high number of false positives relative to true positives. Mohan et al. (2025) reported a lower sensitivity of 68%, suggesting more missed events, while Azadi et al. (2024) reported only a validation accuracy of 87%, which measures the overall proportion of correctly classified instances in a held-out validation set, without distinguishing between false positives and false negatives.

The majority of these methods adopt a modular architecture, using object detection models to crop relevant video regions (e.g., around the ball or players), which are then passed to a separate action classification model. For example, Rezaei and Wu (2022) used a YOLO-based ball tracker to crop image patches, which were then fed into a temporal shift module (TSM) (Lin et al., 2019) with a ResNet-50 backbone (He et al., 2015). Similarly, Azadi et al. (2024) and Mohan et al. (2025) implemented player or head detection to crop and isolate regions of interest for classification. By contrast, Giancola et al. (2023) developed a fully end-to-end approach using uncropped broadcast video and popular architectures such as NetVLAD++ (Giancola and Ghanem, 2021) and PTS (Hong et al., 2022) for direct action spotting. These models differ substantially in complexity. The ResNet-50 backbone used in Rezaei and Wu (2022) includes around 25 million trainable parameters, making it computationally expensive to train from scratch. In contrast, Azadi et al. (2024) used a more compact long-term recurrent convolutional network (LRCN) (Donahue et al., 2016) with an estimated 100,000 parameters, while Mohan et al. (2025) describe a small 3D CNN of unspecified size. These simpler architectures offer advantages in deployment, albeit potentially at the cost of detection performance. Transfer learning and fine-tuning of pretrained models, such as ResNet-50 trained on the widely used Kinetics human action dataset (Kay et al., 2017), were employed by Rezaei and Wu (2022) to reduce the computational cost of training a large network from scratch and to leverage knowledge learned from similar datasets.

In all cases discussed here, training has relied on manually annotated datasets of ground truth head impacts, often sourced from elite sport broadcast footage. This not only raises ethical and legal considerations for researchers (e.g., content rights), but also presents a highly labour-intensive bottleneck preceding model training. For example, the dataset used by Rezaei and Wu (2022) consisted of 4,843 manually annotated head contact events, while (Azadi et al., 2024) manually annotated 150 events, later augmented to 600.

The issues discussed here highlight the practical importance of reusing pretrained models and aligning with existing open-source efforts where possible. For instance, the latest release of the SoccerNet action spotting challenge includes annotated headers (Cioppa et al., 2024), offering a useful benchmark for association football-specific HAE detection tasks. Such datasets can serve as a foundation for fine-tuning rather than full re-training of models from scratch, significantly reducing the development burden for prospective models in the field of HAE research.

### 4.3 Pose estimation

As stated earlier, with the focus of this review being on quantitative video analysis methods from which kinematic parameters can be extracted, an area of significant interest in the field of HAE study is that of pose estimation (Edwards et al., 2021; Tierney, 2021). In the computer vision field, the goal of the pose estimation task is to detect the position and orientation of a person or an object. By definition, this objective therefore aligns closely with the goals of videogrammetric techniques such as MBIM, and to a lesser extent, point tracking, which were discussed in Section 3.

Pose estimation can offer deeper biomechanical insight into HAEs than simple action detection approaches, as it enables the tracking of body segment kinematics throughout an event. In general, pose estimation involves predicting the 2D or 3D positions of anatomical landmarks across video frames, and can be applied to reconstruct full-body or joint-specific motion dynamics. Yuan et al. (2024a), Yuan et al. (2024b) used pose estimation to model the dynamics of skiing and other fall events, while Gildea et al. (2024) applied similar techniques to reconstruct cyclist crash kinematics. In rugby, pose tracking has been used to quantify joint motion during staged tackles (Blythman et al., 2022) and to inform injury risk classification models based on tackle biomechanics (Martin et al., 2021; Nonaka et al., 2022). To date, pose estimation accuracy in the context of HAE analysis has only been evaluated in a controlled laboratory setting by Blythman et al. (2022). In that study, a pre-trained 3D pose estimation model (Iskakov et al., 2019) achieved “out-of-the-box” mean per-joint position errors (the average Euclidean distance between predicted and ground truth joint locations, from marker-based motion capture) of approximately 47 mm . Importantly, however, no studies have thus far attempted a direct comparison between such markerless motion capture systems and the traditional approaches like MBIM discussed in Section 3. As a result, it remains unclear how their relative accuracies compare in practice.

Several studies have identified the potential of pose estimation for head acceleration event (HAE) analysis (Edwards et al., 2021; Tierney, 2021; Rezaei and Wu, 2022); however, systematic validation of these methods in real-world sports settings using large-scale ground truth pose datasets remains in its early stages. Efforts such as the WorldPose dataset (Jiang et al., 2024), used to benchmark monocular pose estimation in broadcast football footage, mark important progress toward closing this gap (albeit in a related but distinct domain to HAEs). The dataset includes 88 association football video sequences with approximately 2.5 million ground truth full-body poses. Notably, constructing such a dataset is significantly more complex than those developed for action detection models. As part of their annotation process, Jiang et al. (2024) used multi-camera bounding box tracking, initial 2D and 3D pose estimation, followed by bootstrapping and bundle adjustment processes to correct inaccuracies in the initial predictions, particularly those caused by occlusions or poor visibility. As a result, developing a comparable dataset specifically for HAE analysis would demand substantial financial resources and manual effort.

In parallel, practical deployment of player tracking and pose estimation in real-world sports settings is increasingly being explored through systems that rely on lower-quality, monocular video. For example, commercial tools such as Spiideo offer cloud-based player tracking using fixed-position broadcast-style cameras, which are already deployed in many elite and semi-professional sporting environments. Similarly, UAV-based videogrammetry has shown promise in capturing full-body kinematics from single or multiple aerial camera views, even in outdoor, unstructured environments. Approaches such as DroCap (Zhou et al., 2018) and FlyCap (Xu et al., 2016) have demonstrated that drone-mounted cameras can also be used to reconstruct 3D human pose. Despite indicating the potential for low-cost player tracking and pose estimation with more flexible camera setups, these methods come with additional technical and regulatory challenges that could further limit their widespread adoption in HAE contexts.

Furthermore, a key limitation in using many of the generic human pose estimation models for head tracking lies in their representation of the head. Many models treat the head as a single point or segment rigidly linked to the neck/torso without resolving its independent motion, which limits their ability to resolve full six degrees of freedom motion. Advances in parametric human mesh models, such as HybrIK (Li et al., 2022), HybrIK-X (Li et al., 2023), and GLAMR (Yuan et al., 2022), enable more detailed recovery of local head motion, though often still rely on coarse body-level cues. More precise results have been achieved using dedicated head pose estimation networks that directly regress six degree of freedom pose from cropped head regions (Hempel et al., 2022; 2024; Martyniuk et al., 2022; Cobo et al., 2024). A recent benchmark by Kupyn et al. (2024) showed that specialised head pose models outperformed full-body estimators on challenging sequences, supporting their adoption for accurate video-based head motion recovery. Hasija and Takhounts (2022) bypassed the need for explicit head pose estimation by using a deep learning model (combining CNNs and LSTMs) to directly predict head kinematics from frames of simulated crash videos, achieving correlation coefficients for predicted peak angular velocities of 0.73, 0.85, and 0.92 for X,Y, and Z components, respectively. The model was, however, trained and evaluated on entirely synthetic data under ideal conditions, which limits its applicability to real-world scenarios.

### 4.4 Video quality enhancement

An avenue which potentially presents greater actionability in the near-term is in the use of deep learning for various video quality enhancement processes, with Stark N. E.-P. et al. (2025) noting the potential benefit of using advanced algorithms, such as motion deblurring techniques or deep learning-based video enhancement, as a preliminary step to mitigate the effects of motion blur in MBIM analysis.

Deep learning-based motion deblurring and denoising methods offer a promising avenue to improve low-quality footage for quantitative analysis of HAEs. Models such as DeblurGAN-v2 (Kupyn et al., 2019) and Restormer (Zamir et al., 2022) have shown strong performance in recovering detail from motion-blurred video sequences. More generally, efforts have also addressed the task of increasing the spatial (pixel) resolution of low-resolution images and videos. For example, ESRGAN (Wang et al., 2018) and Real-ESRGAN (Wang X. et al., 2021) use generative adversarial networks to enhance image resolution while preserving realistic textures and sharpness. Video-specific approaches such as BasicVSR++ (Chan et al., 2021) extend this capability to temporal sequences, maintaining consistency across frames. These spatial enhancement techniques may prove particularly useful in scenarios requiring precise localisation of visual features, such as helmet markings, facial keypoints, or anatomical joint centres for landmark tracking and model alignment. However, it is important to note that these models do not recover true resolution in a physical sense. Instead, they infer plausible high-frequency content based on learned priors from their training data, and should therefore be applied with caution, particularly in relation to footage with characteristics that differ substantially from the original training data of the model.

In the wider field of biomechanics, there is also growing interest in the use of deep learning methods to enhance the temporal resolution of video, representing a potentially delimiting factor in the analysis of low sample rate video footage. Although such techniques have not yet seen widespread application in HAE analysis, they have shown promise in related domains. For example, Einfalt et al. (2023) demonstrated that transformer-based models can perform temporal upsampling, learning to upsample low-frame-rate pose data and thereby reconstruct kinematics at finer temporal scales. Similarly, Dunn et al. (2023) applied video frame interpolation to recover sub-frame motion details in the context of human gait analysis. These approaches could be especially valuable in sports applications, where high-speed impacts often occur between frames, and accurately capturing fine-grained motion dynamics is critical for understanding injury mechanisms and improving predictive modelling.

Together, these video enhancement techniques may serve as valuable preprocessing tools that improve the reliability and accuracy of downstream quantitative methods, especially when analysing HAEs in low quality or existing video datasets, where reshooting is not an option. However, as with any of the deep learning methods discussed throughout this section, it is vital that care is taken, particularly where models are applied in scenarious which differ significantly from their training data.

## 5 Discussion

As shown throughout this review, quantitative video analysis represents a promising yet underutilised approach for the study of HAEs in sport. While wearable sensors remain the primary method for measuring HAE kinematics in the field, they are subject to several limitations, including poor coupling to the skull (Wu et al., 2016b), varying accuracy with proximity of impact to sensor (Le Flao et al., 2025), user discomfort or non-compliance (Roe et al., 2024), and high false-positive rates that necessitate time-consuming manual verification (Patton et al., 2020). Video-based approaches (especially when quantitative rather than qualitative) offer a flexible, scalable, and non-invasive alternative that can supplement or, in certain contexts, replace sensor-based methods by providing estimations of key kinematic parameters associated with HAEs.

An overview of the videogrammetric tools and techniques currently used to extract HAE kinematics from video has been provided, with a particular focus on current capabilities of widely-used point tracking and MBIM methods. These approaches, while validated in controlled environments (Bailey et al., 2018; Tierney et al., 2018a), remain labour-intensive and limited by the frame rate and camera coverage of the source footage. As such, their application to routine, “in-the-wild” (i.e., real-world, uncontrolled settings outside of a laboratory) sports analysis remains limited in practice. MBIM pipelines typically require hours of manual effort per impact (Tierney et al., 2019), as a result of typically requiring manual model alignment required across multiple camera views, thus constraining their scalability in large datasets or real-time settings.

Recent developments in deep learning and computer vision present significant opportunities to overcome these limitations. Markerless pose estimation models (Zheng et al., 2023), specialised head pose networks (Asperti and Filippini, 2023), and action detection pipelines (Giancola et al., 2023; Rezaei and Wu, 2022) now enable the automatic detection and tracking of head, body and object motion directly from video, including broadcast footage. To potentially improve accuracy of both traditional and deep learning approaches, additional video quality enhancement steps leveraging modern deep learning algorithms may be of use. Methods for temporal upsampling (Einfalt et al., 2023) also offer the potential to recover motion signals at frame rates that exceed the source video, opening the door to higher-fidelity kinematic reconstruction in scenarios where camera sampling rate would otherwise be a limiting factor.

Despite these advances, several key challenges remain. Many current pose estimation models still treat the head as a single point or segment rigidly linked to the neck/torso without resolving its independent motion, which limits their ability to resolve full six degrees of freedom motion. Second, the generalisability of these models to real-world, high-occlusion sports environments remains an open question; many pose estimation networks are trained on clean, lab-style datasets with well-lit scenes and unobstructed views. Third, there is a lack of standardised evaluation protocols for validating these models in the context of HAE biomechanics. Efforts to incorporate quantitative video measures into workflows in which downstream predictions of brain injury metrics, such as peak angular acceleration, or predicted brain strain (Zhan et al., 2021) are obtained remain in their infancy (Yuan et al., 2024a; Karton et al., 2025), and thus their accuracy on a large scale remains to be demonstrated.

To realise the full potential of video-based HAE analysis, several future research directions are recommended:

• Domain specific benchmark datasets: Publicly available video datasets with ground-truth kinematic data and action labels, following the precedent set by other sports research efforts such as WorldPose (Jiang et al., 2024) and SoccerNet (Deliege et al., 2021), within the HAE research domain would facilitate fair comparison of quantitative video-based methods.

• Domain-specific models: Pose and head tracking models trained or fine-tuned on sports video (especially those involving collisions, occlusions, and rapid motion) will likely outperform generic “out-of-the-box” models.

• End-to-end pipelines: Complete end-to-end pipelines combining action detection, pose estimation, wearable sensors, and brain strain prediction could lead to practical tools for automated, scalable analysis of HAEs (either in real-time or retrospectively).

• Standardised reporting: Adoption of reporting frameworks such as the CHAMP checklist (Arbogast et al., 2022) across both traditional and machine learning-based video analysis pipelines will improve reproducibility and allow more meaningful cross-study comparison.

In summary, it appears that quantitative video analysis has the potential to transition from a labour-intensive supplementary tool to a viable method for measuring, reconstructing, and modelling HAEs in sport. Realising this potential, however, will require continued methodological development and rigorous validation efforts. With these advances, video-based approaches could play a central role in large-scale exposure surveillance, retrospective concussion analysis, and, ultimately, real-time decision support to enhance athlete safety.

## References

[B1] Abdel-Aziz Y. I. Karara H. M. Hauck M. (2015). Direct linear Transformation from Comparator coordinates into object space coordinates in Close-range Photogrammetry. Photogrammetric Eng. and Remote Sens. 81, 103–107. 10.14358/PERS.81.2.103

[B2] Arbogast K. B. Caccese J. B. Buckley T. A. McIntosh A. S. Henderson K. Stemper B. D. (2022). Consensus head acceleration measurement practices (CHAMP): Origins, methods, transparency and disclosure. Ann. Biomed. Eng. 50, 1317–1345. 10.1007/s10439-022-03025-9 35920964 PMC9652170

[B3] Arrué P. Toosizadeh N. Babaee H. Laksari K. (2020). Low-rank representation of head impact kinematics: a data-driven Emulator. Front. Bioeng. Biotechnol. 8, 555493. 10.3389/fbioe.2020.555493 33102454 PMC7546353

[B4] Asperti A. Filippini D. (2023). Deep learning for head pose estimation: a survey. SN Comput. Sci. 4, 349. 10.1007/s42979-023-01796-z

[B5] Azadi A. Dehghan P. Graham R. Hoshizaki B. (2024). “A deep learning network for detecting head impacts in ice hockey from 2D Game video,” in *Proceedings. International IRCOBI Conference on the Biomechanics of* impacts (Stockholm, Sweden).

[B6] Bailey A. Funk J. Lessley D. Sherwood C. Crandall J. Neale W. (2018). Validation of a videogrammetry technique for analysing American football helmet kinematics. Sports Biomech. 19, 678–700. 10.1080/14763141.2018.1513059 30274537

[B7] Bailey A. M. Sherwood C. P. Funk J. R. Crandall J. R. Carter N. Hessel D. (2020). Characterization of concussive events in professional American football using videogrammetry. Ann. Biomed. Eng. 48, 2678–2690. 10.1007/s10439-020-02637-3 33025319

[B8] Bailly N. Llari M. Donnadieu T. Masson C. Arnoux P. J. (2017). Head impact in a snowboarding accident. Scand. J. Med. and Sci. Sports 27, 964–974. 10.1111/sms.12699 27185578

[B9] Basinas I. McElvenny D. M. Pearce N. Gallo V. Cherrie J. W. (2022). A systematic review of head impacts and acceleration associated with soccer. Int. J. Environ. Res. Public Health 19, 5488. 10.3390/ijerph19095488 35564889 PMC9100160

[B10] Belson K. (2013). N.F.L. Agrees to Settle concussion Suit for $765 million. The New York Times.

[B11] Bian K. Mao H. (2020). Mechanisms and variances of rotation-induced brain injury: a parametric investigation between head kinematics and brain strain. Biomechanics Model. Mechanobiol. 19, 2323–2341. 10.1007/s10237-020-01341-4 32449073

[B12] Blythman R. Saxena M. Tierney G. J. Richter C. Smolic A. Simms C. (2022). Assessment of deep learning pose estimates for sports collision tracking. J. Sports Sci. 40, 1885–1900. 10.1080/02640414.2022.2117474 36093680

[B13] [Dataset] Bogo F. Kanazawa A. Lassner C. Gehler P. Romero J. Black M. J. (2016). Keep it SMPL: automatic estimation of 3D human pose and shape from a single image. 561, 578. 10.1007/978-3-319-46454-1_34

[B14] Bull A. (2024). Rugby brain injury lawsuit stuck in legal limbo – and players are still suffering. Guardian. Available online at: https://www.theguardian.com/sport/2024/oct/20/rugby-brain-injury-lawsuit-stuck-in-legal-limbo-and-players-are-still-suffering#:∼:text=But%20those%20players%20are%20still,to%20properly%20begin%20before%202026 .

[B15] Butterfield J. P. Andrew K. Clara R. Michael A. Hoshizaki T. B. (2023). A video analysis examination of the frequency and type of head impacts for player positions in youth ice hockey and FE estimation of their impact severity. Sports Biomech. 0, 1–17. 10.1080/14763141.2023.2186941 36911883

[B16] Campolettano E. T. Gellner R. A. Rowson S. (2018). “Relationship between impact velocity and resulting head accelerations during head impacts in youth football,” in Proceedings. International IRCOBI Conference on the biomechanics of impacts. PMC6760664 31555774

[B17] Cazzola D. Holsgrove T. P. Preatoni E. Gill H. S. Trewartha G. (2017). Cervical spine injuries: a Whole-body Musculoskeletal model for the analysis of Spinal loading. PLOS ONE 12, e0169329. 10.1371/journal.pone.0169329 28052130 PMC5214544

[B18] Chan K. C. K. Zhou S. Xu X. Loy C. C. (2021). BasicVSR++: improving video Super-resolution with enhanced Propagation and alignment. 10.48550/arXiv.2104.13371

[B19] Chen P. Y. Chou L. S. Hu C. J. Chen H. H. (2015). “Finite element simulations of brain responses to soccer-heading impacts,” in 1st global Conference on biomedical engineering and 9th Asian-Pacific Conference on medical and biological engineering. Editors Su F.-C. Wang S.-H. Yeh M.-L. (Cham: Springer International Publishing), 118–119. 10.1007/978-3-319-12262-5_33

[B20] Chen W. P. Andrew K. Clara G. Michael D. Michael R. Hoshizaki T. B. (2023). A comparison of frequency and magnitude of head impacts between Pee Wee and Bantam youth ice hockey. Sports Biomech. 22, 728–751. 10.1080/14763141.2020.1754450 32538288

[B21] Choi W. J. Wakeling J. M. Robinovitch S. N. (2015). Kinematic analysis of video-captured falls experienced by older adults in long-term care. J. Biomechanics 48, 911–920. 10.1016/j.jbiomech.2015.02.025 25769730

[B22] Cioppa A. Giancola S. Somers V. Joos V. Magera F. Held J. (2024). SoccerNet 2024 challenges results. 10.48550/arXiv.2409.10587

[B23] Clark J. M. Taylor K. Post A. Hoshizaki T. B. Gilchrist M. D. (2018). Comparison of ice hockey goaltender helmets for concussion type impacts. Ann. Biomed. Eng. 46, 986–1000. 10.1007/s10439-018-2017-7 29600424

[B24] Clark J. M. Adanty K. Post A. Hoshizaki T. B. Clissold J. McGoldrick A. (2020a). Proposed injury thresholds for concussion in equestrian sports. J. Sci. Med. Sport 23, 222–236. 10.1016/j.jsams.2019.10.006 31690492

[B25] Clark J. M. Hoshizaki T. B. Annaidh A. N. Gilchrist M. D. (2020b). Equestrian helmet standards: do they represent real-world accident conditions? Ann. Biomed. Eng. 48, 2247–2267. 10.1007/s10439-020-02531-y 32399843

[B26] Clark J. M. A. Kevin P. Andrew H. T. Blaine A. Ni A. Gilchrist M. D. (2021). A parametric analysis of factors that determine head injury outcomes following equestrian fall accidents. Int. J. Crashworthiness 26, 295–308. 10.1080/13588265.2019.1705694

[B27] Cobo A. Valle R. Buenaposada J. M. Baumela L. (2024). On the representation and methodology for wide and short range head pose estimation. Pattern Recognit. 149, 110263. 10.1016/j.patcog.2024.110263

[B28] Colyer S. L. Evans M. Cosker D. P. Salo A. I. T. (2018). A review of the evolution of vision-based motion analysis and the integration of advanced computer vision methods towards developing a markerless system. Sports Med. - Open 4, 24–15. 10.1186/s40798-018-0139-y 29869300 PMC5986692

[B29] Corazza S. Mündermann L. Gambaretto E. Ferrigno G. Andriacchi T. P. (2010). Markerless motion capture through visual hull, Articulated ICP and subject specific model generation. Int. J. Comput. Vis. 87, 156–169. 10.1007/s11263-009-0284-3

[B30] Daneshvar D. H. Nowinski C. J. McKee A. C. Cantu R. C. (2011). The Epidemiology of sport-related concussion. Clin. Sports Med. 30, 1–17. 10.1016/j.csm.2010.08.006 21074078 PMC2987636

[B31] Daniel R. W. Rowson S. Duma S. M. (2012). Head impact exposure in youth football. Ann. Biomed. Eng. 40, 976–981. 10.1007/s10439-012-0530-7 22350665 PMC3310979

[B32] Deliege A. Cioppa A. Giancola S. Seikavandi M. J. Dueholm J. V. Nasrollahi K. (2021). “SoccerNet-v2: a dataset and benchmarks for Holistic understanding of broadcast soccer videos,” in *2021 IEEE/CVF Conference on computer Vision and* pattern recognition Workshops (CVPRW) (Nashville, TN, USA: IEEE), 4503–4514. 10.1109/CVPRW53098.2021.00508

[B33] Denny-Brown D. Russell W. R. (1941). Experimental Cerebral concussion. Brain 64, 93–164. 10.1093/brain/64.2-3.93 PMC1394062 16995229

[B34] Dixit P. Liu G. R. (2017). A review on recent development of finite element models for head injury simulations. Archives Comput. Methods Eng. 24, 979–1031. 10.1007/s11831-016-9196-x

[B35] Donahue J. Hendricks L. A. Rohrbach M. Venugopalan S. Guadarrama S. Saenko K. (2016). Long-term recurrent convolutional networks for visual recognition and description. 10.48550/arXiv.1411.4389 27608449

[B36] Dunn M. Wheat J. Miller S. Haake S. Goodwill S. (2012). “Reconstructing 2D planar coordinates using linear and nonlinear techniques,” in ISBS-conference proceedings archive.

[B37] Dunn M. Kennerley A. Murrell-Smith Z. Webster K. Middleton K. Wheat J. (2023). Application of video frame interpolation to markerless, single-camera gait analysis. Sports Eng. 26, 22. 10.1007/s12283-023-00419-3

[B38] Edwards S. Lee R. Fuller G. Buchanan M. Tahu T. Tucker R. (2021). 3D biomechanics of rugby tackle techniques to inform future rugby research practice: a systematic review. Sports Med. - Open 7, 39. 10.1186/s40798-021-00322-w 34097146 PMC8184906

[B39] Einfalt M. Ludwig K. Lienhart R. (2023). “Uplift and upsample: Efficient 3D human pose estimation with uplifting transformers,” in *2023 IEEE/CVF winter Conference on Applications of computer vision (WACV)* (waikoloa, HI, USA: IEEE), 2902–2912. 10.1109/WACV56688.2023.00292

[B40] England R. (2025). Advanced biofidelic headforms for high fidelity Re-Enactment of real-world head impacts in cricket. Thesis: Loughborough University. 10.26174/thesis.lboro.28323377.v1

[B41] Farmer J. Mitchell S. Sherratt P. Miyazaki Y. (2022). A human surrogate neck for traumatic brain injury research. Front. Bioeng. Biotechnol. 10, 854405. 10.3389/fbioe.2022.854405 36601390 PMC9806148

[B42] Förstner W. Wrobel B. P. (2016). *Photogrammetric computer vision*, vol. 11 of *Geometry and computing* . Cham: Springer International Publishing. 10.1007/978-3-319-11550-4

[B43] Frechede B. McIntosh A. (2007). “Use of MADYMO’s human facet model to evaluate the risk of head injury in impact,” in 20th International technical Conference on the enhanced safety of Vehicles (ESV)National Highway Traffic safety administration.

[B44] Fréchède B. Mcintosh A. S. (2009). Numerical reconstruction of real-Life concussive football impacts. Med. and Sci. Sports and Exerc. 41, 390–398. 10.1249/MSS.0b013e318186b1c5 19127185

[B45] Funk J. R. McIntosh A. S. Withnall C. Wonnacott M. Jadischke R. (2022). Best practices for conducting physical reconstructions of head impacts in sport. Ann. Biomed. Eng. 50, 1409–1422. 10.1007/s10439-022-03024-w 35876938

[B46] Gabler L. F. Crandall J. R. Panzer M. B. (2018). Development of a metric for predicting brain strain responses using head kinematics. Ann. Biomed. Eng. 46, 972–985. 10.1007/s10439-018-2015-9 29594689

[B47] Gabler L. F. Crandall J. R. Panzer M. B. (2019). Development of a Second-order system for rapid estimation of maximum brain strain. Ann. Biomed. Eng. 47, 1971–1981. 10.1007/s10439-018-02179-9 30515603

[B48] Gabler L. F. Huddleston S. H. Dau N. Z. Lessley D. J. Arbogast K. B. Thompson X. (2020). On-field performance of an instrumented mouthguard for detecting head impacts in American football. Ann. Biomed. Eng. 48, 2599–2612. 10.1007/s10439-020-02654-2 33078368

[B49] Gabler L. Patton D. Begonia M. Daniel R. Rezaei A. Huber C. (2022). Consensus head acceleration measurement practices (CHAMP): laboratory validation of wearable head kinematic devices. Ann. Biomed. Eng. 50, 1356–1371. 10.1007/s10439-022-03066-0 36104642 PMC9652295

[B50] Ghazi K. Wu S. Zhao W. Ji S. (2021). Instantaneous Whole-brain strain estimation in dynamic head impact. J. Neurotrauma 38, 1023–1035. 10.1089/neu.2020.7281 33126836 PMC8054523

[B51] Giancola S. Ghanem B. (2021). “Temporally-aware feature Pooling for action spotting in soccer broadcasts,” in 2021 IEEE/CVF Conference on computer vision and pattern recognition Workshops (CVPRW), 4485–4494doi. 10.1109/CVPRW53098.2021.00506

[B52] Giancola S. Cioppa A. Georgieva J. Billingham J. Serner A. Peek K. (2023). “Towards active learning for action spotting in association football videos,” in *2023 IEEE/CVF C*onference on computer Vision and pattern recognition Workshops (CVPRW) (Vancouver, BC, Canada: IEEE), 5098–5108. 10.1109/CVPRW59228.2023.00538

[B53] Gibson T. Shewchenko N. Withnall C. (1995). “Biofidelity improvements to the Hybrid III neck,” in Proceedings OF the FOURTEENTH INTERNATIONAL technical CONFERENCE ON ENHANCED safety OF VEHICLES, 94-S1-O–13.

[B54] Gildea K. Hall D. Cherry C. R. Simms C. (2024). Forward dynamics computational modelling of a cyclist fall with the inclusion of protective response using deep learning-based human pose estimation. J. Biomechanics 163, 111959. 10.1016/j.jbiomech.2024.111959 38286096

[B55] Goodin P. Gardner A. J. Dokani N. Nizette B. Ahmadizadeh S. Edwards S. (2021). Development of a machine-learning-based classifier for the identification of head and body impacts in elite level Australian rules football players. Front. Sports Act. Living 3, 725245. 10.3389/fspor.2021.725245 34870193 PMC8640084

[B56] Greenwald R. M. Gwin J. T. Chu J. J. Crisco J. J. (2008). Head impact severity measures for evaluating Mild traumatic brain injury risk exposure. Neurosurgery 62, 789–798. 10.1227/01.neu.0000318162.67472.ad 18496184 PMC2790598

[B57] Gruen A. (1997). Fundamentals of videogrammetry — a review. Hum. Mov. Sci. 16, 155–187. 10.1016/S0167-9457(96)00048-6

[B58] Gyemi D. L. Andrews D. M. Jadischke R. (2021). Three-dimensional video analysis of helmet-to-ground impacts in North American youth football. J. Biomechanics 125, 110587. 10.1016/j.jbiomech.2021.110587 34274559

[B59] Gyemi D. L. Jadischke R. Andrews D. M. (2023). Validation of a multi-camera videogrammetry approach for quantifying helmet impact velocity in football. Sports Eng. 26, 31. 10.1007/s12283-023-00423-7

[B60] Hajiaghamemar M. Margulies S. S. (2021). Multi-scale white Matter Tract Embedded brain finite element model predicts the location of traumatic Diffuse axonal injury. J. Neurotrauma 38, 144–157. 10.1089/neu.2019.6791 32772838 PMC7757550

[B61] Hajiaghamemar M. Wu T. Panzer M. B. Margulies S. S. (2020). Embedded axonal fiber tracts improve finite element model predictions of traumatic brain injury. Biomechanics Model. Mechanobiol. 19, 1109–1130. 10.1007/s10237-019-01273-8 PMC7203590 31811417

[B62] Hartley R. Zisserman A. (2004). Multiple view geometry in computer vision. 2 edn. Cambridge: Cambridge University Press. 10.1017/CBO9780511811685

[B63] Hasija V. Takhounts E. G. (2022). Deep learning methodology for predicting time history of head angular kinematics from simulated crash videos. Sci. Rep. 12, 6526. 10.1038/s41598-022-10480-w 35444174 PMC9021239

[B64] He K. Zhang X. Ren S. Sun J. (2015). Deep residual learning for image recognition. 10.48550/arXiv.1512.03385

[B65] Hedrick T. L. (2008). Software techniques for two- and three-dimensional kinematic measurements of biological and biomimetic systems. Bioinspiration and Biomimetics 3, 034001. 10.1088/1748-3182/3/3/034001 18591738

[B66] Hempel T. Abdelrahman A. A. Al-Hamadi A. (2022). “6D rotation representation for unconstrained head pose estimation,” in 2022 IEEE International Conference on image processing (ICIP), 2496–2500. 10.1109/ICIP46576.2022.9897219

[B67] Hempel T. Abdelrahman A. A. Al-Hamadi A. (2024). Toward robust and Unconstrained full range of rotation head pose estimation. IEEE Trans. Image Process. 33, 2377–2387. 10.1109/TIP.2024.3378180 38512742

[B68] Hendricks S. K. Fred N. Lambert M. (2012). Velocity and acceleration before contact in the tackle during rugby union matches. J. Sports Sci. 30, 1215–1224. 10.1080/02640414.2012.707328 22853045

[B69] Hochreiter S. Schmidhuber J. (1997). Long short-term memory. Neural Comput. 9, 1735–1780. 10.1162/neco.1997.9.8.1735 9377276

[B70] Hodgson V. R. (1975). National Operating Committee on standards for athletic equipment football helmet certification program. Med. Sci. Sports 7, 225–232. 10.1249/00005768-197500730-00023 1207436

[B71] Holbourn A. H. S. (1943). Mechanics of head injuries. Lancet 242, 438–441. 10.1016/S0140-6736(00)87453-X

[B72] Hong J. Zhang H. Gharbi M. Fisher M. Fatahalian K. (2022). Spotting temporally precise, fine-grained events in video. 33, 51. 10.1007/978-3-031-19833-5_3

[B73] Hubbard R. P. McLeod D. G. (1974). Definition and development of A crash Dummy head. SAE Tech. Pap. 741193, SAE Int. Warrendale, PA, 741193. 10.4271/741193

[B74] Ionescu C. Papava D. Olaru V. Sminchisescu C. (2014). Human3.6M: large scale datasets and predictive methods for 3D human sensing in natural environments. IEEE Trans. pattern analysis Mach. Intell. 36, 1325–1339. 10.1109/TPAMI.2013.248 26353306

[B75] Iskakov K. Burkov E. Lempitsky V. Malkov Y. (2019). Learnable triangulation of human pose. 7717, 7726. 10.1109/iccv.2019.00781

[B76] Jadischke R. Zendler J. Lovis E. Elliott A. Goulet G. (2019). “Development of a methodology and preliminary analysis of head impacts in American 7-v-7 non-tackle football,” in *Proceedings of the IRCO*BI Conference (Florence, Italy).

[B77] Jadischke R. Zendler J. Lovis E. Elliott A. Goulet G. C. (2020). Quantitative and qualitative analysis of head and body impacts in American 7v7 non-tackle football. BMJ Open Sport and Exerc. Med. 6, e000638. 10.1136/bmjsem-2019-000638 PMC7011012 32095268

[B78] Ji S. Zhao W. Li Z. McAllister T. W. (2014). Head impact accelerations for brain strain-related responses in contact sports: a model-based investigation. Biomechanics Model. Mechanobiol. 13, 1121–1136. 10.1007/s10237-014-0562-z PMC5621926 24610384

[B79] Ji S. Ghajari M. Mao H. Kraft R. H. Hajiaghamemar M. Panzer M. B. (2022). Use of brain biomechanical models for monitoring impact exposure in contact sports. Ann. Biomed. Eng. 50, 1389–1408. 10.1007/s10439-022-02999-w 35867314 PMC9652195

[B80] Jiang T. Billingham J. Müksch S. Zarate J. Evans N. Oswald M. R. (2024). “WorldPose: a world Cup dataset for global 3D human pose estimation,” in Computer vision – ECCV 2024: 18th European Conference, milan, Italy, September 29–October 4, 2024, proceedings, Part XIX (Berlin, Heidelberg: Springer-Verlag), 343–362. 10.1007/978-3-031-72655-2_20

[B81] Johnson K. Chowdhury S. Lawrimore W. Mao Y. Mehmani A. Prabhu R. (2016). Constrained topological optimization of a football helmet facemask based on brain response. Mater. and Des. 111, 108–118. 10.1016/j.matdes.2016.08.064

[B82] Jones B. Tooby J. Weaving D. Till K. Owen C. Begonia M. (2022). Ready for impact? A validity and feasibility study of instrumented mouthguards (iMGs). Br. J. Sports Med. 56, 1171–1179. 10.1136/bjsports-2022-105523 35879022

[B83] Kaplan D. (2021). NHL paid $70.6 million in legal fees for concussion settlement that paid players $18.49 million. The New York Times.

[B84] Karton C. Blaine Hoshizaki T. Gilchrist M. D. (2020). A novel repetitive head impact exposure measurement tool differentiates player position in National Football League. Sci. Rep. 10, 1200. 10.1038/s41598-019-54874-9 31992719 PMC6987098

[B85] Karton C. Post A. Laflamme Y. Kendall M. Cournoyer J. Robidoux M. A. (2021). Exposure to brain trauma in six age divisions of minor ice hockey. J. Biomechanics 116, 110203. 10.1016/j.jbiomech.2020.110203 33412437

[B86] Karton C. Hoshizaki T. B. Graham R. Ross G. Clouthier A. (2025). System and method for monitoring brain trauma exposure

[B87] [Dataset] Kay W. Carreira J. Simonyan K. Zhang B. Hillier C. Vijayanarasimhan S. (2017). The Kinetics human action video dataset. 10.48550/arXiv.1705.06950

[B88] Kendall M. Oeur A. Brien S. E. Cusimano M. Marshall S. Gilchrist M. D. (2020). Accident reconstructions of falls, collisions, and punches in sports. J. Concussion 4, 2059700220936957. 10.1177/2059700220936957

[B89] Kenny R. Elez M. Clansey A. Virji-Babul N. Wu L. C. (2024). Individualized monitoring of longitudinal heading exposure in soccer. Sci. Rep. 14, 1796. 10.1038/s41598-024-52163-8 38245604 PMC10799858

[B90] Kern J. Lober T. Hermsdörfer J. Endo S. (2022). A neural network for the detection of soccer headers from wearable sensor data. Sci. Rep. 12, 18128. 10.1038/s41598-022-22996-2 36307512 PMC9616946

[B91] King D. Hume P. Gissane C. Brughelli M. Clark T. (2016). The influence of head impact threshold for reporting data in contact and collision sports: systematic review and original data analysis. Sports Med. 46, 151–169. 10.1007/s40279-015-0423-7 26545363

[B92] Kleiven S. (2013). Why most traumatic brain injuries are not caused by linear acceleration but skull Fractures are. Front. Bioeng. Biotechnol. 1, 15. 10.3389/fbioe.2013.00015 25022321 PMC4090913

[B93] Kosziwka G. Champoux L. Cournoyer J. Gilchrist M. Hoshizaki T. (2021). Risk of head injury associated with distinct head impact events in elite women’s hockey. J. Concussion 5, 20597002211058894. 10.1177/20597002211058894

[B94] Krbavac B. P. Cutler J. Lowther S. Karton C. Post A. Robidoux M. (2024). Comparing frequency and maximum principal strain of head impacts for U15 ice hockey leagues with standard and modified body contact rules. J. Biomechanics 176, 112370. 10.1016/j.jbiomech.2024.112370 39423482

[B95] Krizhevsky A. Sutskever I. Hinton G. E. (2012). ImageNet classification with deep convolutional neural networks. Adv. Neural Inf. Process. Syst. 25. 10.1145/3065386

[B96] Krosshaug T. Bahr R. (2005). A model-based image-matching technique for three-dimensional reconstruction of human motion from uncalibrated video sequences. J. Biomechanics 38, 919–929. 10.1016/j.jbiomech.2004.04.033 15713313

[B97] Kuo C. Wu L. Loza J. Senif D. Anderson S. C. Camarillo D. B. (2018). Comparison of video-based and sensor-based head impact exposure. PLOS ONE 13, e0199238. 10.1371/journal.pone.0199238 29920559 PMC6007917

[B98] Kuo C. Patton D. Rooks T. Tierney G. McIntosh A. Lynall R. (2022). On-field deployment and validation for wearable devices. Ann. Biomed. Eng. 50, 1372–1388. 10.1007/s10439-022-03001-3 35960418 PMC9652208

[B99] Kupyn O. Martyniuk T. Wu J. Wang Z. (2019). DeblurGAN-v2: deblurring (Orders-of-Magnitude) faster and better. 10.48550/arXiv.1908.03826

[B100] [Dataset] Kupyn O. Khvedchenia E. Rupprecht C. (2024). VGGHeads: 3D multi head alignment with a large-scale synthetic dataset. Available online at: https://arxiv.org/abs/2407.18245v2.

[B101] Laksari K. Fanton M. Wu L. C. Nguyen T. H. Kurt M. Giordano C. (2020). Multi-directional dynamic model for traumatic brain injury detection. J. Neurotrauma 37, 982–993. 10.1089/neu.2018.6340 31856650 PMC7175617

[B102] Laurentini A. (1994). The visual hull concept for silhouette-based image understanding. IEEE Trans. Pattern Analysis Mach. Intell. 16, 150–162. 10.1109/34.273735

[B103] Le Flao E. Siegmund G. P. Borotkanics R. (2022). Head impact research using inertial sensors in sport: a systematic review of methods, Demographics, and factors contributing to exposure. Sports Med. 52, 481–504. 10.1007/s40279-021-01574-y 34677820

[B104] Le Flao E. Lenetsky S. Siegmund G. P. Borotkanics R. (2024). Capturing head impacts in boxing: a video-based comparison of three wearable sensors. Ann. Biomed. Eng. 52, 270–281. 10.1007/s10439-023-03369-w 37728812

[B105] Le Flao E. Siegmund G. P. Lenetsky S. Borotkanics R. (2025). Quality issues in kinematic traces from three head impact sensors in boxing: Prevalence, effects, and implications for exposure assessment. Ann. Biomed. Eng. 53, 658–672. 10.1007/s10439-024-03647-1 39625630

[B106] Lecun Y. Bottou L. Bengio Y. Haffner P. (1998). Gradient-based learning applied to document recognition. Proc. IEEE 86, 2278–2324. 10.1109/5.726791

[B107] LeCun Y. Bengio Y. Hinton G. (2015). Deep learning. Nature 521, 436–444. 10.1038/nature14539 26017442

[B108] Li J. Xu C. Chen Z. Bian S. Yang L. Lu C. (2022). HybrIK: a Hybrid Analytical-neural Inverse kinematics solution for 3D human pose and shape estimation. 10.48550/arXiv.2011.14672 40031204

[B109] Li J. Bian S. Yang L. Lu C. (2023). HybrIK-X: Hybrid Analytical-neural Inverse kinematics for Whole-body mesh recovery. 10.48550/arXiv.2304.05690 40031204

[B110] Lin J. Gan C. Han S. (2019). “TSM: temporal shift module for efficient video understanding,” in 2019 IEEE/CVF International Conference on computer vision (ICCV), 7082–7092. 10.1109/ICCV.2019.00718

[B111] Linendoll K. (2013). Could X2’s skin patch detect concussions? Available online at: https://www.espn.com/blog/playbook/tech/post/_/id/3547/could-x2s-skin-patch-detect-concussions.

[B112] Livingston G. Huntley J. Sommerlad A. Ames D. Ballard C. Banerjee S. (2020). Dementia prevention, intervention, and care: 2020 report of the Lancet Commission. Lancet 396, 413–446. 10.1016/S0140-6736(20)30367-6 32738937 PMC7392084

[B113] Luna T. (2013). Mass. companies team up to prevent head injuries - the Boston Globe. Available online at: https://www.bostonglobe.com/business/2013/07/14/reebok-introduce-concussion-sensor-for-sports/s7L509zIM5lBrHXb0ylDDM/story.html.

[B114] Mackay D. F. Russell E. R. Stewart K. MacLean J. A. Pell J. P. Stewart W. (2019). Neurodegenerative disease Mortality among former professional soccer players. N. Engl. J. Med. 381, 1801–1808. 10.1056/NEJMoa1908483 31633894 PMC8747032

[B115] Madhukar A. Ostoja-Starzewski M. (2019). Finite element methods in human head impact simulations: a review. Ann. Biomed. Eng. 47, 1832–1854. 10.1007/s10439-019-02205-4 30693442

[B116] Marchand E. Uchiyama H. Spindler F. (2016). Pose estimation for augmented reality: a Hands-on survey. IEEE Trans. Vis. Comput. Graph. 22, 2633–2651. 10.1109/TVCG.2015.2513408 26731768

[B117] Martin Z. Hendricks S. Patel A. (2021). “Automated tackle injury risk assessment in contact-based sports - a rugby union example,” in Proceedings of the IEEE/CVF Conference on computer vision and pattern recognition, 4594–4603.

[B118] Martyniuk T. Kupyn O. Kurlyak Y. Krashenyi I. Matas J. Sharmanska V. (2022). DAD-3DHeads: a large-scale dense, accurate and diverse dataset for 3D head alignment from a single image. 20910, 20920. 10.1109/cvpr52688.2022.02027

[B119] McGill K. (2022). Investigation into brain and Intracranial structure representations in FE head models 10.7488/era/2544

[B120] McGill K. Teixeira-Dias F. Callanan A. (2020). A review of validation methods for the Intracranial response of FEHM to Blunt impacts. Appl. Sci. 10, 7227. 10.3390/app10207227

[B121] McIntosh A. S. McCrory P. Cromerford J. (2000). The dynamics of concussive head impacts in rugby and Australian rules football. Med. and Sci. Sports and Exerc. 32, 1980. 10.1097/00005768-200012000-00002 11128839

[B122] McIntosh A. S. Patton D. A. Fréchède B. Pierré P.-A. Ferry E. Barthels T. (2014). The biomechanics of concussion in unhelmeted football players in Australia: a case–control study. BMJ Open 4, e005078. 10.1136/bmjopen-2014-005078 PMC4039841 24844272

[B123] McKee A. Cantu R. Nowinski C. Hedley-Whyte E. Gavett B. Budson A. (2009). Chronic traumatic encephalopathy in athletes: progressive tauopathy after repetitive head injury. J. Neuropathology Exp. Neurology 68, 709–735. 10.1097/NEN.0b013e3181a9d503 PMC2945234 19535999

[B124] Meliambro J. K. Clara C. Janie P. Andrew H. Blaine T. Gilchrist M. D. (2022). Comparison of head impact frequency and magnitude in youth tackle football and ice hockey. Comput. Methods Biomechanics Biomed. Eng. 25, 936–951. 10.1080/10255842.2021.1987420 34615414

[B125] Michio Clark J. Post A. Blaine Hoshizaki T. Gilchrist M. D. (2018). Distribution of brain strain in the Cerebrum for laboratory impacts to ice hockey Goaltender Masks. J. Biomechanical Eng. 140, 121007. 10.1115/1.4040605 30029266

[B126] Mohan M. Weaving D. Gardner A. J. Hendricks S. Stokes K. A. Phillips G. (2025). Can a novel computer vision-based framework detect head-on-head impacts during a rugby league tackle? Inj. Prev.–2023-045129. 10.1136/ip-2023-045129 39832883

[B127] Motiwale S. Eppler W. Hollingsworth D. Hollingsworth C. Morgenthau J. Kraft R. H. (2016). “Application of neural networks for filtering non-impact transients recorded from biomechanical sensors,” in 2016 IEEE-EMBS International Conference on biomedical and Health Informatics (BHI), 204–207. 10.1109/BHI.2016.7455870

[B128] Neale W. Held J. S. Jadischke R. Rundell S. Arrington D. (2022). Video analysis of head acceleration events.

[B129] Newman J. Beusenberg M. Fournier E. Shewchenko N. Withnall C. King A. (1999). A new biomechanical assessment of Mild traumatic brain injury Part 1 – methodology 10.1016/j.jbiomech.2004.06.025 15922758

[B130] Newman J. Barr C. Beusenberg M. Fournier E. Shewchenko N. Welbourne E. (2000). A new biomechanical assessment of Mild traumatic brain injury Part 2 – results and conclusions.

[B131] Nguyen J. V. K. Brennan J. H. Mitra B. Willmott C. (2019). Frequency and magnitude of game-related head impacts in male contact sports athletes: a systematic review and Meta-analysis. Sports Med. 49, 1575–1583. 10.1007/s40279-019-01135-4 31175636

[B132] Nonaka N. Fujihira R. Nishio M. Murakami H. Tajima T. Yamada M. (2022). “End-to-End high-risk tackle detection system for rugby,” in *2022 IEEE/CVF Conference on computer Vision and pattern recognition Workshops (CVPRW)* (new Orleans, LA, USA: IEEE), 3549–3558. 10.1109/CVPRW56347.2022.00399

[B133] O’Connor K. L. Rowson S. Duma S. M. Broglio S. P. (2017). Head-impact–measurement devices: a systematic review. J. Athl. Train. 52, 206–227. 10.4085/1062-6050.52.2.05 28387553 PMC5384819

[B134] O’Keeffe E. Kelly E. Liu Y. Giordano C. Wallace E. Hynes M. (2020). Dynamic Blood-brain barrier regulation in Mild traumatic brain injury. J. Neurotrauma 37, 347–356. 10.1089/neu.2019.6483 31702476 PMC10331162

[B135] Patton D. A. (2016). A review of instrumented equipment to investigate head impacts in sport. Appl. Bionics Biomechanics 2016, 1–16. 10.1155/2016/7049743 PMC4993933 27594780

[B136] Patton D. A. Huber C. M. Jain D. Myers R. K. McDonald C. C. Margulies S. S. (2020). Head impact sensor studies in sports: a systematic review of exposure confirmation methods. Ann. Biomed. Eng. 48, 2497–2507. 10.1007/s10439-020-02642-6 33051746 PMC7674240

[B137] Pellman E. J. Viano D. C. Tucker A. M. Casson I. R. Waeckerle J. F. (2003). Concussion in professional football: reconstruction of game impacts and injuries. Neurosurgery 53, 799–814. 10.1093/neurosurgery/53.3.799 14519212

[B138] Perkins R. A. Bakhtiarydavijani A. Ivanoff A. E. Jones M. Hammi Y. Prabhu R. K. (2022). Assessment of brain injury biomechanics in soccer heading using finite element analysis. Brain Multiphysics 3, 100052. 10.1016/j.brain.2022.100052

[B139] Post A. Clark J. M. Robertson D. G. E. Hoshizaki T. B. Gilchrist M. D. (2017). The effect of acceleration signal processing for head impact numeric simulations. Sports Eng. 20, 111–119. 10.1007/s12283-016-0219-5

[B140] Post A. Koncan D. Kendall M. Cournoyer J. Michio Clark J. Kosziwka G. (2018). Analysis of speed accuracy using video analysis software. Sports Eng. 21, 235–241. 10.1007/s12283-018-0263-4

[B141] Post A. H. Blaine T. Dawson L. Cournoyer J. Clara C. (2019a). The biomechanics of concussion for ice hockey head impact events. Comput. Methods Biomechanics Biomed. Eng. 22, 631–643. 10.1080/10255842.2019.1577827 30829543

[B142] Post A. Karton C. Robidoux M. Gilchrist M. D. Hoshizaki T. B. (2019b). An examination of the brain trauma in Novice and Midget ice hockey: implications for helmet innovation. *CMBES* Proc. 42.

[B143] Post A. K. Clara T. Odette H. Blaine T. Gilchrist M. D. (2021). Comparison of frequency and magnitude of head impacts experienced by Peewee boys and girls in games of youth ice hockey. Comput. Methods Biomechanics Biomed. Eng. 24, 1–13. 10.1080/10255842.2020.1805442 32787715

[B144] Raymond S. J. Cecchi N. J. Alizadeh H. V. Callan A. A. Rice E. Liu Y. (2022). Physics-informed machine learning improves detection of head impacts. Ann. Biomed. Eng. 50, 1534–1545. 10.1007/s10439-022-02911-6 35303171

[B145] Rezaei A. Wu L. C. (2022). Automated soccer head impact exposure tracking using video and deep learning. Sci. Rep. 12, 9282. 10.1038/s41598-022-13220-2 35661123 PMC9166706

[B146] Roe G. Whitehead S. Starling L. Allan D. Cross M. Falvey É. (2024). Embracing the impact from instrumented mouthguards (iMGs): a survey of iMG managers’ perceptions of staff and player interest into the technology, data and barriers to use. Eur. J. Sport Sci. 24, 670–681. 10.1002/ejsc.12101 38874970 PMC11235837

[B147] Rotundo M. P. Sokol-Randell D. Bleakley C. Donnelly P. Tierney G. (2023). Characteristics of potential concussive events in elite hurling: a video-analysis study. Ir. J. Med. Sci. (1971 -) 192, 3175–3185. 10.1007/s11845-023-03307-8 PMC10692028 36800054

[B148] Rowson S. Duma S. M. (2013). Brain injury prediction: assessing the combined Probability of concussion using linear and rotational head acceleration. Ann. Biomed. Eng. 41, 873–882. 10.1007/s10439-012-0731-0 23299827 PMC3624001

[B149] Rowson S. Mihalik J. Urban J. Schmidt J. Marshall S. Harezlak J. (2022). Consensus head acceleration measurement practices (CHAMP): study design and statistical analysis. Ann. Biomed. Eng. 50, 1346–1355. 10.1007/s10439-022-03101-0 36253602 PMC9652215

[B150] Russell E. R. Mackay D. F. Stewart K. MacLean J. A. Pell J. P. Stewart W. (2021). Association of field position and career length with risk of neurodegenerative disease in male former professional soccer players. JAMA Neurol. 78, 1057–1063. 10.1001/jamaneurol.2021.2403 34338724 PMC8329793

[B151] Russell E. R. Mackay D. F. Lyall D. Stewart K. MacLean J. A. Robson J. (2022). Neurodegenerative disease risk among former international rugby union players. J. Neurology, Neurosurg. and Psychiatry 93, 1262–1268. 10.1136/jnnp-2022-329675 PMC9669247 36195436

[B152] Schonberger J. L. Frahm J.-M. (2016). “Structure-from-Motion Revisited,” in *2016 IEEE Conference on computer Vision and pattern recognition (CVPR)* (Las Vegas, NV, USA: IEEE), 4104–4113. 10.1109/CVPR.2016.445

[B153] Sharma P. K. Smith L. (2024). A comparison of soccer heading scenarios on the brain 10.17028/rd.lboro.27051337.v1

[B154] Sherwood C. P. Grogan F. McMurry T. L. Funk J. R. Crandall J. R. Sills A. (2025). Tackle techniques and characteristics associated with a concussion in tackling players in the National football league. Am. J. Sports Med. 53, 1142–1151. 10.1177/03635465251321005 40037391

[B155] Shishov N. Elabd K. Komisar V. Chong H. Robinovitch S. N. (2021). Accuracy of Kinovea software in estimating body segment movements during falls captured on standard video: effects of fall direction, camera perspective and video calibration technique. PLOS ONE 16, e0258923. 10.1371/journal.pone.0258923 34695159 PMC8544843

[B156] Shotton J. Fitzgibbon A. Cook M. Sharp T. Finocchio M. Moore R. (2011). “Real-time human pose recognition in parts from single depth images,” in Cvpr 2011, 1297–1304. 10.1109/CVPR.2011.5995316

[B157] Stark N. E. P. Henley E. S. Reilly B. A. Kuehl D. R. Rowson S. (2025a). Kinematic insights into older Adult fall-related head impacts: Boundary conditions and injury risk. J. Am. Med. Dir. Assoc. 26, 105545. 10.1016/j.jamda.2025.105545 40088941

[B158] Stark N. E.-P. Henley E. S. Reilly B. A. Nowinski J. S. Ferro G. M. Madigan M. L. (2025b). Uncalibrated single-camera view video tracking of head impact speeds using model-based image matching. Ann. Biomed. Eng. 53, 1359–1369. 10.1007/s10439-025-03705-2 40082330 PMC12075344

[B159] Stewart W. Buckland M. E. Abdolmohammadi B. Affleck A. J. Alvarez V. E. Gilchrist S. (2023). Risk of chronic traumatic encephalopathy in rugby union is associated with length of playing career. Acta Neuropathol. 146, 829–832. 10.1007/s00401-023-02644-3 37872234 PMC10627955

[B160] Sugiyama K. Nakamura H. (1999). A method of de-interlacing with motion compensated interpolation. IEEE Trans. Consumer Electron. 45, 611–616. 10.1109/30.793548

[B161] Szeliski R. (2022). Computer vision: algorithms and applications. Cham: Springer International Publishing. 10.1007/978-3-030-34372-9

[B162] Takahashi Y. Yanaoka T. (2017). “A study of injury criteria for brain injuries in Traffic accidents,” in 25th International technical Conference on the enhanced safety of Vehicles (ESV)National Highway Traffic safety administration.

[B163] Taylor K. Post A. Hoshizaki T. B. Gilchrist M. D. (2019). The effect of a novel impact management strategy on maximum principal strain for reconstructions of American football concussive events. Proc. Institution Mech. Eng. Part P J. Sports Eng. Technol. 233, 503–513. 10.1177/1754337119857434

[B164] Tierney G. (2021). Concussion biomechanics, head acceleration exposure and brain injury criteria in sport: a review. Sports Biomech. 0, 1888–1916. 10.1080/14763141.2021.2016929 34939531

[B165] Tierney G. J. Simms C. K. (2017). The effects of tackle height on inertial loading of the head and neck in Rugby Union: a multibody model analysis. Brain Inj. 31, 1925–1931. 10.1080/02699052.2017.1385853 29064724

[B166] Tierney G. J. Simms C. (2019). Predictive Capacity of the MADYMO multibody human body model applied to head kinematics during rugby union tackles. Appl. Sci. 9, 726. 10.3390/app9040726

[B167] Tierney G. J. Joodaki H. Krosshaug T. Forman J. L. Crandall J. R. Simms C. K. (2018a). Assessment of model-based image-matching for future reconstruction of unhelmeted sport head impact kinematics. Sports Biomech. 17, 33–47. 10.1080/14763141.2016.1271905 28632058

[B168] Tierney G. J. Richter C. Denvir K. Simms C. K. (2018b). Could lowering the tackle height in rugby union reduce ball carrier inertial head kinematics? J. Biomechanics 72, 29–36. 10.1016/j.jbiomech.2018.02.023 29525242

[B169] Tierney G. J. Gildea K. Krosshaug T. Simms C. K. (2019). Analysis of ball carrier head motion during a rugby union tackle without direct head contact: a case study. Int. J. Sports Sci. and Coach. 14, 190–196. 10.1177/1747954119833477

[B170] Tierney G. J. Power J. Simms C. (2021). Force experienced by the head during heading is influenced more by speed than the mechanical properties of the football. Scand. J. Med. and Sci. Sports 31, 124–131. 10.1111/sms.13816 32881107

[B171] Tooby J. Woodward J. Tucker R. Jones B. Falvey É. Salmon D. (2024). Instrumented mouthguards in elite-level Men’s and women’s rugby union: the incidence and Propensity of head acceleration events in matches. Sports Med. 54, 1327–1338. 10.1007/s40279-023-01953-7 37906425 PMC11127838

[B172] Vale A. P. Andrew C. Janie H. Blaine T. Gilchrist M. D. (2022). Influence of play type on the magnitude and number of head impacts sustained in youth American football. Comput. Methods Biomechanics Biomed. Eng. 25, 1195–1210. 10.1080/10255842.2021.2003345 34788175

[B173] Vaswani A. Shazeer N. Parmar N. Uszkoreit J. Jones L. Gomez A. N. (2017). Attention is all you need. Adv. Neural Inf. Process. Syst. 30. 10.5555/3295222.3295349

[B174] Wang H. Kläser A. Schmid C. Liu C.-L. (2011). “Action recognition by dense trajectories,” in Cvpr 2011, 3169–3176. 10.1109/CVPR.2011.5995407

[B175] Wang X. Yu K. Wu S. Gu J. Liu Y. Dong C. (2018). ESRGAN: Enhanced Super-resolution generative adversarial networks. 10.48550/arXiv.1809.00219

[B176] Wang T. Kenny R. Wu L. C. (2021a). Head impact sensor Triggering Bias introduced by linear acceleration thresholding. Ann. Biomed. Eng. 49, 3189–3199. 10.1007/s10439-021-02868-y 34622314

[B177] Wang X. Xie L. Dong C. Shan Y. (2021b). Real-ESRGAN: training real-world Blind Super-resolution with pure synthetic data. 1905, 1914. 10.1109/iccvw54120.2021.00217

[B178] Wdowicz D. Ptak M. (2023). Numerical approaches to Pedestrian impact simulation with human body models: a review. Archives Comput. Methods Eng. 30, 4687–4709. 10.1007/s11831-023-09949-2

[B179] Withnall C. Shewchenko N. Gittens R. Dvorak J. (2005). Biomechanical investigation of head impacts in football. Br. J. Sports Med. 39, i49–i57. 10.1136/bjsm.2005.019182 16046356 PMC1765309

[B180] Woodward J. Jones B. Phillips G. Till K. Hendricks S. Tucker R. (2025). Tackle characteristics resulting in potential head injuries in elite Men’s rugby league: a video analysis study of 746 tackles. Eur. J. Sport Sci. 25, e12270. 10.1002/ejsc.12270 39999225 PMC11855370

[B181] Wu L. (2020). Sports concussions: can head impact sensors help biomedical engineers to design better headgear? Br. J. Sports Med. 54, 370–371. 10.1136/bjsports-2019-101300 31810973

[B182] WuL. C.LaksariK.KuoC.LuckJ. F.KleivenS.Dale’ BassC. R. (2016a). Bandwidth and sample rate requirements for wearable head impact sensors. J. Biomechanics 49, 2918–2924. 10.1016/j.jbiomech.2016.07.004 27497499

[B183] Wu L. C. Nangia V. Bui K. Hammoor B. Kurt M. Hernandez F. (2016b). *In vivo* evaluation of wearable head impact sensors. Ann. Biomed. Eng. 44, 1234–1245. 10.1007/s10439-015-1423-3 26289941 PMC4761340

[B184] Wu L. C. Kuo C. Loza J. Kurt M. Laksari K. Yanez L. Z. (2017). Detection of American football head impacts using biomechanical features and support vector machine classification. Sci. Rep. 8, 855. 10.1038/s41598-017-17864-3 29321637 PMC5762632

[B185] Wu S. Zhao W. Rowson B. Rowson S. Ji S. (2020). A network-based response feature matrix as a brain injury metric. Biomechanics Model. Mechanobiol. 19, 927–942. 10.1007/s10237-019-01261-y PMC7210066 31760600

[B186] Wu S. Zhao W. Ji S. (2022). Real-time dynamic simulation for highly accurate spatiotemporal brain deformation from impact. Comput. methods Appl. Mech. Eng. 394, 114913. 10.1016/j.cma.2022.114913 35572209 PMC9097909

[B187] Xu L. Fang L. Cheng W. Guo K. Zhou G. Dai Q. (2016). FlyCap: markerless motion capture using multiple Autonomous Flying cameras. 10.48550/arXiv.1610.09534 28727553

[B188] Yamazaki J. Gilgien M. Kleiven S. Mcintosh A. S. Nachbauer W. Müller E. (2015). Analysis of a Severe head injury in world Cup Alpine skiing. Med. and Sci. Sports and Exerc. 47, 1113–1118. 10.1249/MSS.0000000000000511 25207934

[B189] Yeadon M. Challis J. (1994). The future of performance-related sports biomechanics research. J. Sports Sci. 12, 3–32. 10.1080/02640419408732156 8158746

[B190] Yuan Y. Iqbal U. Molchanov P. Kitani K. Kautz J. (2022). GLAMR: global occlusion-Aware human mesh recovery with dynamic cameras. 11028, 11039. 10.1109/cvpr52688.2022.01076

[B191] Yuan Q. Li X. Zhou Z. Kleiven S. (2024a). A novel framework for video-informed reconstructions of sports accidents: a case study correlating brain injury pattern from multimodal neuroimaging with finite element analysis. Brain Multiphysics 6, 100085. 10.1016/j.brain.2023.100085

[B192] Yuan Q. Lindgren N. Li X. Kleiven S. (2024b). End-to-End workflow for finite element accident reconstruction: coupling video-based human pose estimation with HBM

[B193] Zamir S. W. Arora A. Khan S. Hayat M. Khan F. S. Yang M.-H. (2022). Restormer: Efficient transformer for high-resolution image Restoration. 10.48550/arXiv.2111.09881

[B194] Zhan X. Li Y. Liu Y. Domel A. G. Alizadeh H. V. Raymond S. J. (2021). The relationship between brain injury criteria and brain strain across different types of head impacts can be different. J. R. Soc. Interface 18, 20210260. 10.1098/rsif.2021.0260 34062102 PMC8169213

[B195] Zhan X. Li Y. Liu Y. Cecchi N. J. Raymond S. J. Zhou Z. (2023). Machine-learning-based head impact subtyping based on the spectral densities of the measurable head kinematics. J. Sport Health Sci. 12, 619–629. 10.1016/j.jshs.2023.03.003 36921692 PMC10466194

[B196] Zhan X. Liu Y. Cecchi N. J. Callan A. A. Le Flao E. Gevaert O. (2024a). AI-based denoising of head impact kinematics measurements with convolutional neural network for traumatic brain injury prediction. IEEE Trans. Biomed. Eng. 71, 2759–2770. 10.1109/TBME.2024.3392537 38683703

[B197] Zhan X. Liu Y. Cecchi N. J. Towns J. Callan A. A. Gevaert O. (2024b). Identification of head impact locations, speeds, and force based on head kinematics. 10.48550/arXiv.2409.08177 40536866

[B198] Zhang Z. (1999). Flexible camera calibration by viewing a plane from unknown orientations. Proc. Seventh IEEE Int. Conf. Comput. Vis. 1, 666–673 vol.1. 10.1109/ICCV.1999.791289

[B199] Zheng C. Wu W. Chen C. Yang T. Zhu S. Shen J. (2023). Deep learning-based human pose estimation: a survey. ACM Comput. Surv. 56 (11), 1–37. 10.1145/3603618

[B200] Zhou X. Liu S. Pavlakos G. Kumar V. Daniilidis K. (2018). Human motion capture using a drone. 2027, 2033. 10.1109/icra.2018.8462830

[B201] Zimmerman K. A. Cournoyer J. Lai H. Snider S. B. Fischer D. Kemp S. (2023). The biomechanical signature of loss of consciousness: computational modelling of elite athlete head injuries. Brain 146, 3063–3078. 10.1093/brain/awac485 36546554 PMC10316777

